# Promotion of Bone Morphogenetic Protein Signaling by Tetraspanins and Glycosphingolipids

**DOI:** 10.1371/journal.pgen.1005221

**Published:** 2015-05-15

**Authors:** Zhiyu Liu, Herong Shi, Lindsey C. Szymczak, Taner Aydin, Sijung Yun, Katharine Constas, Arielle Schaeffer, Sinthu Ranjan, Saad Kubba, Emad Alam, Devin E. McMahon, Jingpeng He, Neta Shwartz, Chenxi Tian, Yevgeniy Plavskin, Amanda Lindy, Nimra Amir Dad, Sunny Sheth, Nirav M. Amin, Stephanie Zimmerman, Dennis Liu, Erich M. Schwarz, Harold Smith, Michael W. Krause, Jun Liu

**Affiliations:** 1 Department of Molecular Biology and Genetics, Cornell University, Ithaca, New York, United States of America; 2 Laboratory of Molecular Biology, National Institute of Diabetes and Digestive and Kidney Diseases, Bethesda, Maryland, United States of America; The University of North Carolina at Chapel Hill, United States of America

## Abstract

Bone morphogenetic proteins (BMPs) belong to the transforming growth factor β (TGFβ) superfamily of secreted molecules. BMPs play essential roles in multiple developmental and homeostatic processes in metazoans. Malfunction of the BMP pathway can cause a variety of diseases in humans, including cancer, skeletal disorders and cardiovascular diseases. Identification of factors that ensure proper spatiotemporal control of BMP signaling is critical for understanding how this pathway is regulated. We have used a unique and sensitive genetic screen to identify the plasma membrane-localized tetraspanin TSP-21 as a key new factor in the *C*. *elegans* BMP-like “Sma/Mab” signaling pathway that controls body size and postembryonic M lineage development. We showed that TSP-21 acts in the signal-receiving cells and genetically functions at the ligand-receptor level. We further showed that TSP-21 can associate with itself and with two additional tetraspanins, TSP-12 and TSP-14, which also promote Sma/Mab signaling. TSP-12 and TSP-14 can also associate with SMA-6, the type I receptor of the Sma/Mab pathway. Finally, we found that glycosphingolipids, major components of the tetraspanin-enriched microdomains, are required for Sma/Mab signaling. Our findings suggest that the tetraspanin-enriched membrane microdomains are important for proper BMP signaling. As tetraspanins have emerged as diagnostic and prognostic markers for tumor progression, and TSP-21, TSP-12 and TSP-14 are all conserved in humans, we speculate that abnormal BMP signaling due to altered expression or function of certain tetraspanins may be a contributing factor to cancer development.

## Author Summary

The bone morphogenetic protein (BMP) signaling pathway is required for multiple developmental processes during metazoan development. Various diseases, including cancer, can result from mis-regulation of the BMP pathway. Thus, it is critical to identify factors that ensure proper regulation of BMP signaling. Using the nematode *C*. *elegans*, we have devised a highly specific and sensitive genetic screen to identify new modulators in the BMP pathway. Through this screen, we identified three conserved tetraspanin molecules as novel factors that function to promote BMP signaling in a living organism. We further showed that these three tetraspanins likely form a complex and function together with glycosphingolipids to promote BMP signaling. Recent studies have implicated several tetraspanins in cancer initiation, progression and metastasis in mammals. Our findings suggest that the involvement of tetraspanins in cancer may partially be due to their function in modulating the activity of BMP signaling.

## Introduction

Bone morphogenetic proteins (BMPs) belong to the transforming growth factor β (TGFβ) superfamily of secreted polypeptides that regulate a variety of developmental and homeostatic processes [1, 2]. The TGFβ ligands are synthesized as precursor proteins that can be subsequently processed by proteases [3]. Active TGFβ ligands bind to a heterotetrameric receptor complex composed of type I and type II receptors, leading to the phosphorylation of the type I receptor by the type II receptor. The phosphorylated type I receptor then phosphorylates and activates the receptor-regulated Smads (R-Smads). The activated R-Smads form a complex with common-mediator Smads (Co-Smads) and enter the nucleus to regulate downstream gene expression. Malfunction of the TGFβ pathway can result in numerous somatic and hereditary disorders in humans, including various cancers, bone skeletal disorders, and cardiovascular diseases [4–7]. Multiple levels of regulation ensure proper spatiotemporal activity of TGFβ signaling in the correct cellular context [8–11]. Identifying factors involved in modulating the TGFβ pathway and determining their modes of action in vivo will not only provide valuable insights into our understanding of TGFβ signaling, but may also provide therapeutic targets for the many diseases caused by alterations in TGFβ signaling.

*C*. *elegans*, with its wealth of genetic and molecular tools and its well-defined cell lineage, provides an excellent model system to study the functions and modulation of TGFβ signaling during the development of a whole organism at single-cell resolution. There are at least three TGFβ-related pathways in *C*. *elegans*: one that controls dauer formation, one that regulates axon guidance and cell migration, and a third BMP-like “Sma/Mab” pathway that regulates body size and male tail formation, among its multiple functions [12]. The Sma/Mab pathway includes a BMP-like molecule DBL-1 [13, 14], the type I receptor SMA-6 [15], the type II receptor DAF-4 [16], the R-Smads SMA-2 and SMA-3, and the Co-Smad SMA-4 [17]. Loss-of-function mutations in any component of this pathway will cause small body size and male tail sensory ray formation defects [12].

**Fig 1:**
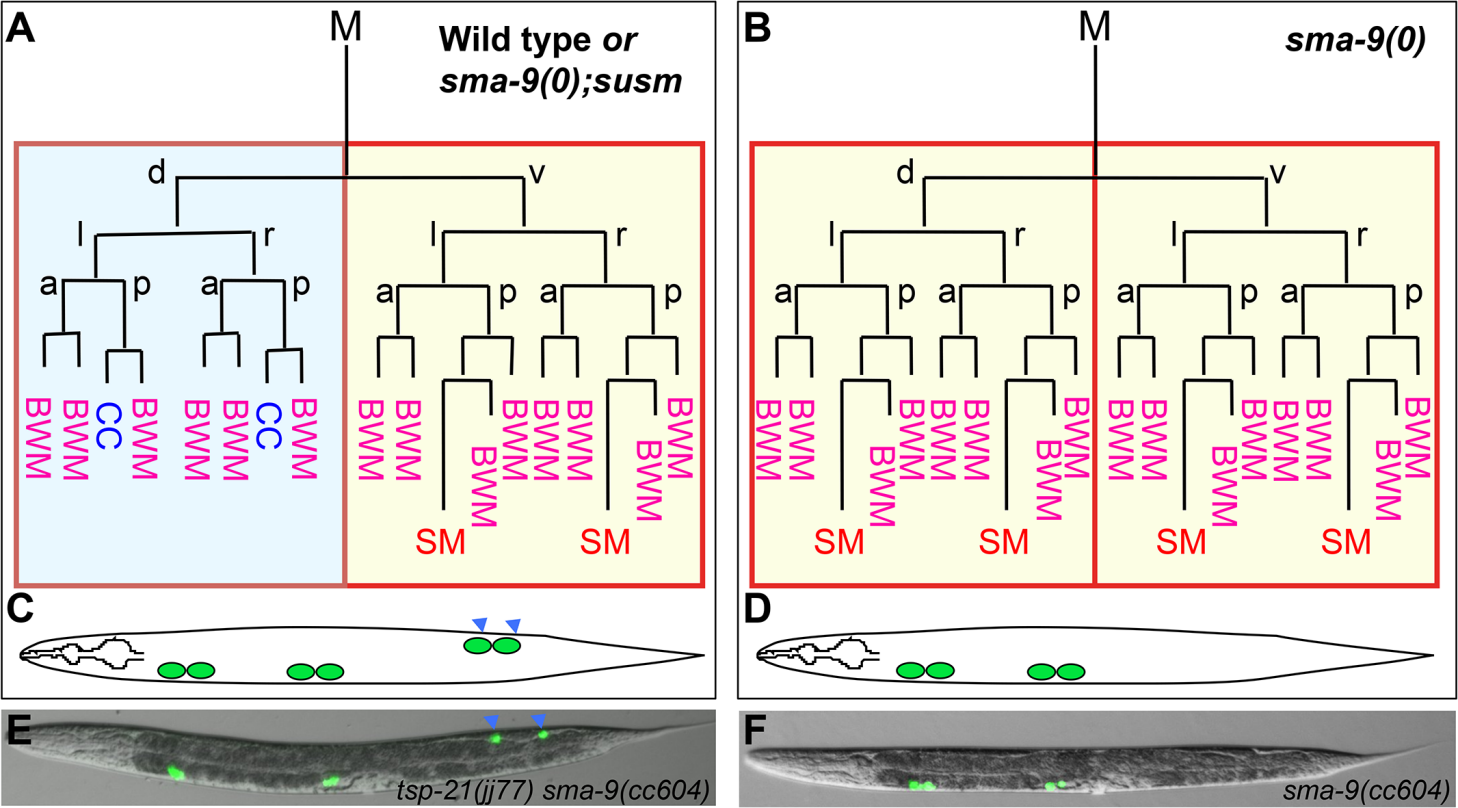
The *sma-9(0)* suppressor mutations revert the M lineage dorsal-to-ventral fate transformation defect in *sma-9(0)* mutants to the wild-type pattern. (A, B) Schematic representation of the M lineage in wild-type or *sma-9(0);susm* (A), and *sma-9(0)* (B) animals. (C-D) Diagrams of an adult wild-type or *sma-9(0);susm* worm (C) and an adult *sma-9(0)* animal (D), showing the CC phenotype. *sma-9(0)* mutants lack the two M-derived CC that are present in wild-type or *sma-9(0);susm* animals (in blue arrowheads). (E and F) Merged GFP and DIC images of *tsp-21(jj77) sma-9(cc604)* (E) and *sma-9(cc604)* (F) worms carrying the *CC*::*gfp* marker at the late L4 stage. BWM: body-wall muscle, CC: coelomocyte, SM: sex myoblast. d: dorsal, v: ventral, l: left, r: right, a: anterior, p: posterior.

**Fig 2:**
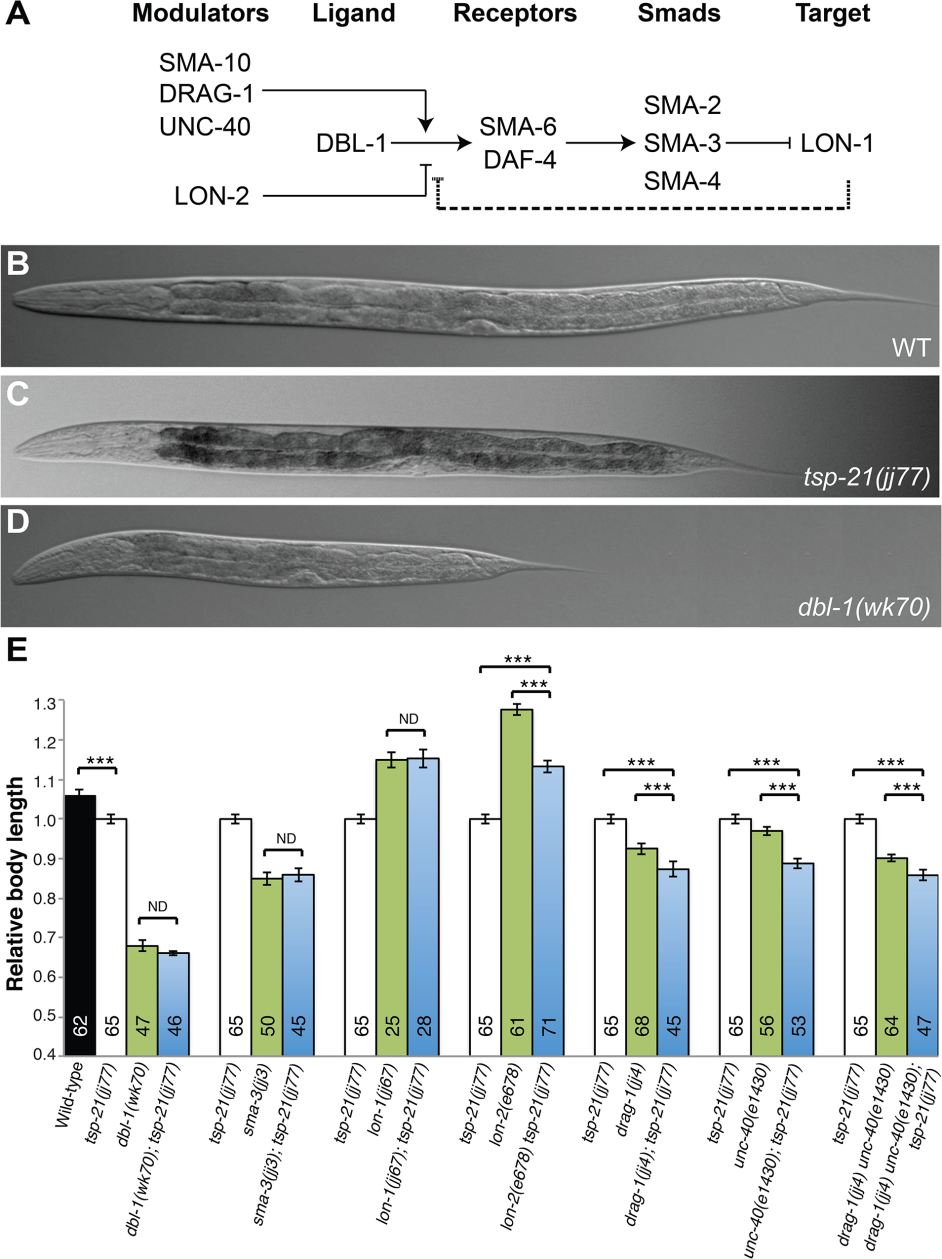
TSP-21 functions at the ligand-receptor level to positively modulate Sma/Mab signaling. (A) The Sma/Mab pathway, showing the core pathway members (DBL-1, SMA-6, DAF-4, SMA-2, 3 and 4) as well as the previously identified positive (SMA-10, DRAG-1 and UNC-40) and negative (LON-2) modulators. We propose that LON-1 also negatively regulates Sma/Mab signaling via a feedback mechanism (dashed line). (B-D) DIC images showing the body size of wild type (B), *tsp-21(jj77)* (C) and *dbl-1(wk70)* (D) worms at the Christmas tree stage of vulval development. (E) Relative body lengths of stage-matched wild-type and various mutant worms measured at the Christmas tree stage. *** *p*<0.0001, (unpaired two-tailed Student’s *t*-test). Error bars represent 95% confidence intervals for the normalized body length. ND, not different.

We have previously shown that the Sma/Mab pathway also plays a role in patterning the *C*. *elegans* postembryonic mesoderm. The hermaphrodite postembryonic mesodermal M lineage arises from a single pluripotent precursor cell, the M mesoblast. During larval development, the M mesoblast divides to produce a dorsal lineage that gives rise to striated bodywall muscles (BWMs) and macrophage-like coelomocytes (CCs), as well as a ventral lineage that produces BWMs and the sex muscle precursor cells, the sex myoblasts (SMs) ([18]; Fig 1A and 1C and 1E). This dorsoventral asymmetry is regulated by the *schnurri* homolog *sma-9* [19]. Mutations in *sma-9* lead to a dorsal-to-ventral fate transformation in the M lineage ([20]; Fig 1B and 1D and 1F). We have shown that mutations in the core components of the Sma/Mab pathway (Fig 2A) do not cause any M lineage defect on their own, but they suppress the dorsoventral patterning defects of *sma-9* mutants, suggesting that SMA-9 regulates M lineage dorsoventral patterning by antagonizing Sma/Mab signaling [20]. Using this *sma-9* M lineage suppression phenotype (Fig 1A and 1C and 1E), we have recently identified two new modulators of the Sma/Mab pathway, the RGM protein DRAG-1 and the DCC/neogenin homolog UNC-40, which directly associate with each other to positively regulate Sma/Mab signaling [21, 22, Fig 2A]. We further showed that their functions in modulating BMP signaling are evolutionarily conserved [22, 23].

In this study, we describe our identification and analysis of additional *sma-9* M lineage phenotype suppressors that function in modulating Sma/Mab signaling. One novel modulator is TSP-21, which belongs to a family of transmembrane molecules called tetraspanins [24]. Tetraspanins are a distinct family of integral membrane proteins that have four conserved transmembrane (TM) domains separated by a small extracellular loop (EC1), a small intracellular loop (IL) and a large extracellular loop (EC2). They are known to interact with each other to form homo- and hetero-oligomers, and organize membranes into the so-called tetraspanin-enriched microdomains that are also enriched in cholesterol and glycosphingolipids [24–27]. There are 33 tetraspanins in humans and 21 in the *C*. *elegans* genome. The in vivo functions of most of these tetraspanins are not well understood. Here we provide evidence that TSP-21, the *C*. *elegans* ortholog of human TSPAN4, TSPAN9 and CD53, is localized to the cell membrane and functions positively to regulate Sma/Mab signaling in the signal-receiving cells at the ligand-receptor level. We further show that two additional tetraspanins that belong to the C8 subfamily of tetraspanins, TSP-12 and TSP-14, also function to promote Sma/Mab signaling. TSP-12 and TSP-14 can physically interact with each other, with TSP-21, and with the type I receptor of the Sma/Mab pathway, SMA-6. In addition, we find that mutants defective in glycosphingolipid biosynthesis exhibit defects in Sma/Mab signaling. Collectively, our results provide in vivo evidence supporting the roles of tetraspanins and glycosphingolipid-enriched membrane microdomains in modulating BMP signaling. Finally, we provide evidence that like TSP-12 and TSP-14, which have been previously shown to function in promoting LIN-12/Notch signaling [28], TSP-21 also appears to function in LIN-12/Notch signaling in a cell type-specific manner.

## Results

### Mutations in the BMP-like Sma/Mab pathway specifically suppress the *sma-9* M lineage phenotype

We have previously shown that mutations in all core components of the Sma/Mab pathway, but not the TGFβ-like dauer pathway, can suppress the M lineage phenotype of *sma-9(0)* mutants [20]. Here we show that null mutations in *unc-129*, which encodes a TGFβ-like molecule important for axon guidance [29], or null mutations in genes that regulate body size but do not function in the Sma/Mab pathway, such as the β-spectrin gene *sma-1* [30], or the cuticle collagen gene *lon-3* [31, 32], do not suppress the M lineage phenotype of *sma-9(0)* mutants (Table 1). Similarly, mutations in the Sma/Mab pathway do not suppress the M lineage defect of *let-381(RNAi)*, which also leads to a dorsal-to-ventral fate transformation defect in the M lineage by inactivating the FoxF/FoxC transcription factor LET-381 ([33]; Table 1). In contrast, two deletion alleles of *sma-10*, which encodes a conserved, leucine-rich repeats- and immunoglobulin (Ig)-like domain (LRIG)-containing transmembrane protein that promotes Sma/Mab signaling in regulating body size [34, Fig 2A], do suppress the M lineage phenotype of *sma-9(0)* mutants (Table 1).

**Table 1:** Mutations in known Sma/Mab pathway genes specifically suppress the *sma-9* M lineage phenotype.

Mutation	Degree of suppression when homozygous^a^ (number of worms)	Degree of suppression when heterozygous^b^ (number of worms)
*dbl-1(wk70)*	100% (n = 60)^*c*^	30% (n = 70)
*sma-6(e1482)*	97% (n = 100)^*c*^	0 (n = 47)
*daf-4(m63)*	98% (n = 112)^*c*^	8.06% (n = 62)
*sma-2(e502)*	100% (n>100)^*c*^	2.67% (n = 225)
*sma-3(e491)*	100% (n>100)^*c*^	24.7% (n = 348)
*sma-4(e729)*	100% (n = 61)^*c*^	21.5% (n = 177)
*lon-2(e678)*	88% (n = 305)^*c*^	2.61% (n = 115)
*drag-1(jj4)*	98.5% (n = 201)^*d*^	0 (n = 39)
*unc-40(e1430)*	88.7% (n = 150)^*e*^	ND
*lon-1(e185)*	33.5% (n = 54)	7.44% (n = 121)
*sma-10(wk89)*	48.9% (n = 534)	ND
*sma-10(ok2224)*	57.5% (n = 569)	ND
*crm-1(tm2218)*	0 (n>100)	ND
*crm-1(RNAi) crm-1(tm2218)*	0 (n>200)	ND
*daf-1(m40)*	0 (n>100)^*c*^	ND
*unc-129(ev554)*	0 (n>100)	ND
*unc-129(ev566)*	0 (n>100)	ND
*sma-1(ru18)*	0 (n>100)	ND
*lon-3(ct417)*	0 (n>100)	ND
*let-381(RNAi)* ^*f*^	7.0% (n = 143)^*g*^	-
*let-381(RNAi); sma-3(jj3)* ^*f*^	6.9% (n = 144)^*g*^	-

Suppression was scored based on the re-appearance of M-derived CCs, which are missing in *sma-9(cc604)* mutant. All animals listed in this table are homozygous for *sma-9(cc604)* except in the case of *let-381(RNAi)*.

ND: not determined.

^*a*^ The worms scored are homozygous for *sma-9(cc604)* and homozygous for the mutation indicated.

^*b*^ The worms scored are homozygous for *sma-9(cc604)* and heterozygous for the mutation indicated.

^*c*^ Data from [20]. All mutations are apparent null alleles.

^*d*^ Data from [21]. *jj4* is an apparent null allele of *drag-1*.

^*e*^ Data from [22]. *e1430* is an apparent null allele of *unc-40*.

^*f*^ The worms scored are wild-type worms or *sma-3(jj3)* worms treated with *let-381(RNAi)* post-embryonically (see Materials and methods). *let-381(RNAi)* leads to the loss of M-derived CCs [33].

^*g*^ The numbers shown refer to percentages of worms with 2 M-derived CCs, which likely resulted from inefficient *let-381(RNAi)*.

**Fig 3:**
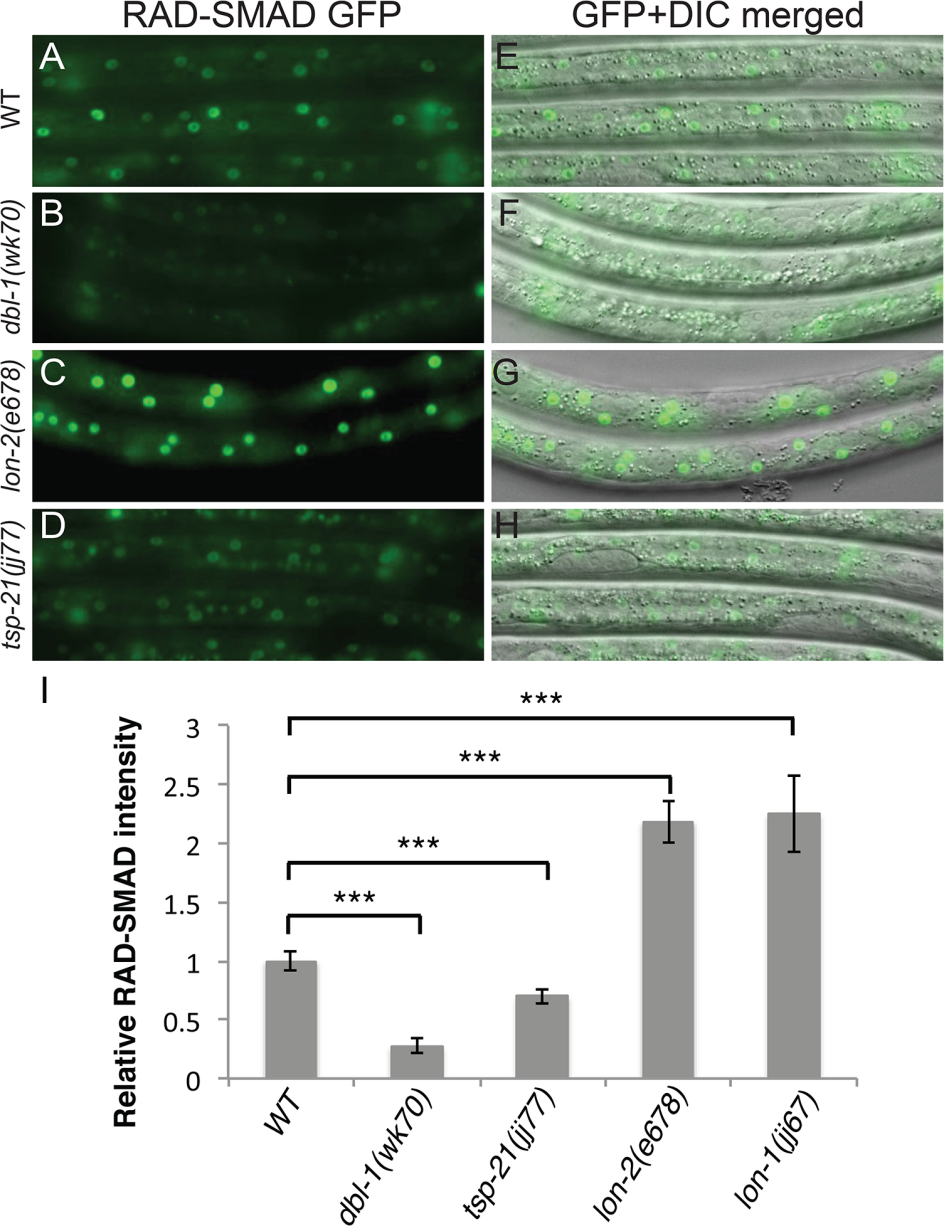
*tsp-21(jj77)* mutants exhibit reduced RAD-SMAD reporter expression. (A-H) Hypodermal expression of the RAD-SMAD GFP reporter in wild type (A, E), *dbl-1(wk70)* (B, F), *lon-2(e678)* (C, G) and *tsp-21(jj77)* (D, H) worms at the L2 stage. The exposure time for all the GFP images was identical. (I) Quantification of the hypodermal RAD-SMAD GFP fluorescence intensity in various mutants compared with wild-type animals (set to 1). *** *p*<0.0001, (unpaired two-tailed Student’s *t*-test). Error bars represent 95% confidence intervals for the normalized RAD-SMAD intensity.

Motivated by the specificity of the BMP-like Sma/Mab pathway mutants in suppressing the *sma-9* M lineage defect and our previous success from the *sma-9* suppressor screen in the identification of evolutionarily conserved modulators of BMP signaling, such as DRAG-1/RGM and UNC-40/DCC/neogenin [21, 22], we performed a large-scale screen for *sma-9* suppressors, named *susm* (suppressor of *sm* *a-9*) mutations, with the aim of identifying additional modulators of BMP signaling (see Materials and Methods). Using a combination of linkage analysis, complementation tests and whole genome sequencing (see Materials and Methods), we identified the corresponding genes for 32 *susm* mutations. As shown in Table 2, our suppressor screen successfully and specifically identified mutations in all core members and known modulators of the Sma/Mab pathway. Intriguingly, we isolated *lon-1(jj67)* as a *sma-9* suppressor and showed that an existing, strong loss-of-function allele, *lon-1(e185)*, also suppresses the *sma-9* M lineage phenotype (Tables 1 and 2). *lon-1* encodes a member of the cysteine-rich secretory protein (CRISP) family of proteins and is known to function downstream of, and be negatively regulated by, the Sma/Mab pathway [13, 35, Fig 2A]. The suppression of the *sma-9* M lineage phenotype by *lon-1* mutations and the increased expression of the Sma/Mab responsive reporter RAD-SMAD [21] in *lon-1(jj67)* mutants (Fig 3B) suggest that LON-1 may exert feedback regulation on Sma/Mab signaling, rather than being strictly regulated by this pathway (Fig 2A). In addition to these factors known to function in Sma/Mab signaling, we also identified a novel factor defined by two non-complementing alleles, *jj60* and *jj77* (Table 2).

**Table 2:** Summary of the mutant alleles isolated in the *sma-9* suppressor screen.

Gene	LG	Protein	Allele	Degree of suppression when homozygous^a^	Degree of suppression when heterozygous^b^	Molecular lesion^p^
*dbl-1*	V	BMP ligand	*jj54* ^*l*^	100% (n = 803)	23.3% (n = 103)	W278Stop (TGG to TGA)
*sma-6*	II	Type I receptor	*jj1* ^*c*^	100% (n = 109)	ND	W328Stop (TGG to TAG)
			*jj69* ^*l*^ ^,^ ^*n*^ ^,^ ^*q*^	94.8% (n = 173)	2% (n = 50)	G to A at -1,886bp
			*jj76* ^*l*^	98.7% (n = 394)	18.99% (n = 79)	W112Stop (TGG to TAG)
*daf-4*	III	Type II receptor	*jj50* ^*m*^	86% (n = 384)	2.63% (n = 38)	A586T (GCA to ACA)
			*jj52* ^*m*^	88% (n = 338)	1.14% (n = 88)	A586T (GCA to ACA)
			*jj53* ^*l*^	87% (n = 162)	ND	M484I (ATG to ATA)
*sma-2*	III	R-SMAD	*jj5* ^*m*^	82.2% (n = 225)	15.2% (n = 79)	G19E (GGA to GAA)
			*jj6* ^*l*^	71% (n = 100)	ND	G19E (GGA to GAA)
			*jj64* ^*l*^	71.2% (n = 139)	3.6% (n = 111)	R67W (CGG to TGG)
			*jj66* ^*l*^	87% (n-710)	ND	C62Y (TGC to TAC)
			*jj72* ^*l*^	96.7% (n = 62)	10% (n = 60)	E100K (GAA to AAA)
*sma-3*	III	R-SMAD	*jj3* ^*c*^	100% (n = 100)	ND	Intron 9 splice donor T to A Truncation after amino acid 318^i^
			*jj55* ^*l*^	100% (n = 703)	12.5% (n = 128)	K265E (AAA to GAA)
			*jj63* ^*l*^	72% (n = 271)	ND	W349Stop (TGG to TAG)
			*jj74* ^*l*^	94.1% (n = 239)	ND	G259E (GGA to GAA)
			*jj75* ^*l*^	100% (n = 173)	ND	Q261Stop (CAG to TAG)
			*jj82* ^*l*^	96.9% (n = 449)	10.94% (n = 64)	G259E (GGA to GAA)
*sma-4*	III	Co-SMAD	*jj51* ^*l*^	100% (n = 1662)	24.49% (n = 49)	S169F (TCT to TTT)
			*jj56*	100% (n = 623)	15.58% (n = 321)	Intron 3 splice donor G to A Truncation after amino acid 155^i^
			*jj70* ^*m*^ ^,^ ^*o*^	97.5% (n = 80)	12.1% (n = 149)	S3330L (TCA to TTA)
			*jj79* ^*l*^	56.2% (n = 539)	15.67% (n = 134)	R261K (AGA to AAA)
*lon-2*	X	Glypican	*jj61* ^*n*^	82.3% (n = 124)	ND	11,781bp deletion after exon 8, Truncation after amino acid 390^j^
*drag-1*	I	RGM	*jj4* ^*d*^	98.5% (N = 201)	0% (n = 39)	Intron 1 splice donor G to A Truncation after amino acid 16^k^
*unc-40*	I	DCC/neogenin	*jj2* ^*m*^	63.3% (n = 128)	24.55% (n = 110)	R677C (CGT to TGT)
			*jj7* ^*m*^	63% (n = 50)	2.48% (n = 121)	R677C (CGT to TGT)
			*jj59* ^*e*^	73.3% (n = 195)	0 (n = 83)	Q628Stop (CAA to TAA)
*sma-10*	IV	LRIG	*jj49* ^*n*^	66% (n = 607)	4.26% (n = 47)	G361R (GGA to AGA)
			*jj57* ^*n*^	95% (n = 1156)	23.19% (n = 111)	Intron 8 splice donor T to A Truncation after amino acid 442^i^
			*jj62* ^*n*^	82.8% (n = 134)	ND	W462Stop (TGG to TGA)
			*jj71* ^*n*^	70.2% (n = 228)	ND	Q593Stop (CAA to TAA)
			*jj73* ^*n*^	88% (n = 188)	5.85% (n = 205)	A434T (GCA to ACA)
			*jj78* ^*n*^	97.1% (n = 615)	12.8% (n = 211)	P643S (CCA to TCA)
			*jj83* ^*n*^	66.2% (n = 757)	7.29% (n = 96)	Y665N (TAT to AAT)
*lon-1*	III	CRISP	*jj67* ^*l*^	66.8% (n = 190)	ND	W197Stop (TGG to TGA)
*tsp-21*	X	Tetraspanin	*jj60* ^*m*^	89.8% (n = 98)	1.04% (n = 96)	G109E (GGA to GAA)
			*jj77* ^*m*^	99.5% (n = 643)	ND	V236E (GTA to GAA) and 84bp deletion of intron 6 into exon 7, 6bp (ATCTCT) insertion 13AA deletion + 2AA insertion^i^
*susm-1* ^*f*^	V		*jj65*	84.3% (n = 255)	11.28% (n = 133)	
			*jj85*	77.9% (n = 240)	4.35% (n = 69)	
			*jj58* ^*g*^	58% (n = 1191)	34.68% (n = 124)	
			*jj68* ^*h*^	23.7% (n = 102)	0 (n = 97)	
			*jj80* ^*h*^	34.6% (n = 81)	ND	
			*jj81* ^*h*^	25.2% (n = 294)	ND	
			*jj84* ^*h*^	23.2% (n = 194)	ND	

Suppression was determined based on the number of M lineage-derived coelomocytes (CCs). *sma-9(cc604)* hermaphrodites have 0 M lineage-derived CCs, while wild-type worms have 2.

ND: not determined

^a^ The worms scored are homozygous for *sma-9(cc604)* and homozygous for the suppressor indicated.

^b^ The worms scored are homozygous for *sma-9(cc604)* and heterozygous for the suppressor indicated.

^c^ Reported in [20].

^d^ Reported in [21].

^e^ Reported in [22].

^f^ Both *jj65* and *jj85* complemented *dbl-1(wk70)* and do not carry any molecular lesions in the *dbl-1* coding or 5’ and 3’ regulatory regions.

^g^ *jj58* might be a dominant *sma-9* suppressor and was not characterized in this study.

^h^ *jj68*, *jj80*, *jj81* and *jj84* were not characterized in this study due to their low degree of suppression of the *sma-9* M lineage phenotype.

^i^ Predicted based on likely splicing defect.

^j^ The predicted truncation at the amino acid level was calculated assuming that the large deletion does not affect the stability of the *lon-2* message in *jj61* animals. The deletion also deletes the majority of the *aman-1* coding region, leaving only 338bp of the *aman-1* first exon to be present. The Susm phenotype is due to the loss of *lon-2* function because *jj61* failed to complement *lon-2(e678)* for the Susm phenotype.

^k^ Aberrant transcripts were confirmed by RT-PCR and reported in [22].

^l^ Corresponding genes were identified via a combination of linkage analysis (linkage to *cup-5(ar465)* III, *sma-9(cc604) X*), snip-SNP mapping [86], and complementation tests based on the Susm phenotype, with the following alleles: *dbl-1(wk70)*, *sma-6(e1482)*, *daf-4(m63)*, *sma-2(e502)*, *sma-3(e491)*, *sma-4(e729)*, *lon-2(e678)*, and *lon-1(e185)*.

^m^ Corresponding genes were identified via WGS (see Materials and methods).

^n^ Corresponding genes were identified via SNP-WGS (see Materials and methods).

^o^ In addition to carrying a mutation in *sma-4*, *jj70* appears to carry another *sma-9* suppressing mutation that maps to LG V and fails to complement *jj65* and *jj85*.

^p^ All molecular lesions were identified or confirmed by Sanger Sequencing.

^q^ A fragment containing 3kb of upstream sequences, the genomic coding region and 2kb of downstream sequences of *sma-6* rescued the Susm phenotype of *jj69*.

### Mutations in the membrane protein tetraspanin-21 (TSP-21) are novel *sma-9* suppressors

**Fig 4:**
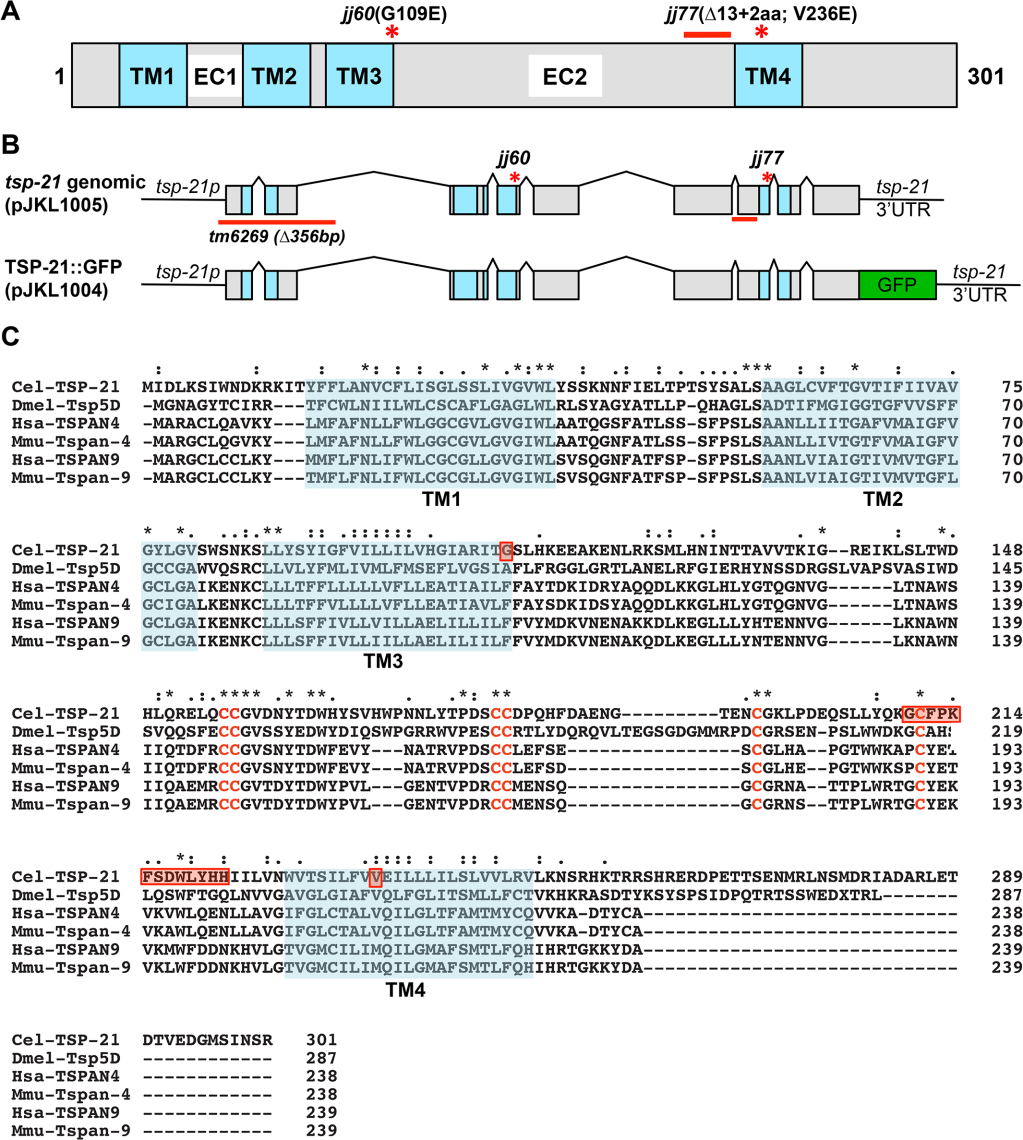
*tsp-21* encodes a conserved tetraspanin protein of the C6a group. (A) A schematic of the TSP-21 protein, showing the four transmembrane (TM) domains and the two extracellular loops (EC1 and EC2). The locations of the *jj60* and *jj77* molecular lesions are shown. (B) Diagrams of the *tsp-21* genomic and GFP tagged constructs. The location of the *tm6269* deletion as well as the *jj60* and *jj77* molecular lesions are shown. (C) A ClustalX sequence alignment [104] of TSP-21 and other C6a tetraspanins from *Drosophila*, human and mouse, highlighting the four transmembrane domains (shaded light green), the conserved cysteine residues (red letters) and the amino acids mutated or deleted in *jj60* or *jj77* (red boxes). Identical residues are marked with asterisks (*) and conserved residues are marked with either colons (:) or periods (.) above the alignment. The Genbank accession numbers for the proteins shown in panel C are: *Drosophila* Tsp5D (isoform C, NP_001259266.1), human TSPAN4 (NP_001020406.1), mouse Tspan-4 (NP_001239517.1), human TSPAN9 (NP_001161792.1), mouse Tspan-9 (NP_780623.1).

We performed whole genome sequencing (WGS) of the novel complementation group that includes *jj60* and *jj77* to identify the corresponding gene. Four lines of evidence indicate that the corresponding gene is *tsp-21*. 1) RNAi of *tsp-21* suppressed the *sma-9* M lineage phenotype (Table 3). 2) Both *jj60* and *jj77* contain molecular lesions in *tsp-21* (Fig 4A): *jj60* contains a G-to-A change in nucleotide 1827, resulting in a Glycine (G) to Glutamic acid (E) change of amino acid 109 (G109E). *jj77* contains a T-to-A change in nucleotide 3327, resulting in a Valine (V) to Glutamic acid (E) change of amino acid 236 (V236E). *jj77* also carries a 84bp deletion (between nucleotides 3202 and 3287) and a 6bp insertion (ATCTCT), resulting in a 13 amino acid deletion and 2 amino acid insertion between amino acids 209 and 223. 3) A DNA fragment containing the genomic sequence of *tsp-21* (5kb upstream, entire coding region including introns, and 1.7kb downstream sequences for C17G1.8 in pJKL1005, Fig 4B) rescued the Susm phenotype of *jj77* (Table 3). 4) A deletion allele of *tsp-21* that we recently obtained, *tm6269*, exhibited defects similar to those of *jj77* and *jj60* animals (Fig 4B and Table 3).

**Table 3:** Suppression of the *sma-9(0)* M lineage defects by *tsp-21* mutations.

Genotype	% of animals with 1–2 M-CCs (total # of animals examined)
Wild-type	100% (n>100)
*sma-9(cc604)*	3% (n = 150)
*tsp-21(RNAi) sma-9(cc604)*	64% (n = 138)
*C41A3*.*1(RNAi) sma-9(cc604)*	0% (n = 150)
*sul-1(RNAi) sma-9(cc604)*	0% (n = 70)
*tsp-21(tm6269)*	100% (n = 483)
*sma-9(RNAi)*	4.4% (n = 635)
*tsp-21(tm6269) sma-9(RNAi)*	98.9% (n = 603)
*tsp-21(jj77)*	100% (n>100)
*tsp-21(jj77) sma-9(cc604)*	85% (n = 517)
*tsp-21(jj77) sma-9(cc604); jjEx[tsp-21p*::*tsp-21 genomic*::*tsp-21 3’UTR]*	2.5% (n = 82)
*tsp-21(jj77) sma-9(cc604); jjIs[tsp-21p*::*tsp-21 genomic*::*gfp*::*tsp-21 3’UTR]*	5% (n = 120)
*tsp-21(jj77) sma-9(cc604); jjEx[hlh-8p*::*tsp-21cDNA]*	4% (n = 385)

*tsp-21* encodes a conserved but previously unstudied 301 amino acid transmembrane protein of the tetraspanin family, TSP-21 (Fig 4C). Based on the number of cysteine (C) residues in the large EC2 loop, tetraspanins can be classified into three groups, C4, C6 and C8 [36, 37]. TSP-21 belongs to the C6a group, with the following configuration of cysteine residues in EC2: CCG——CC——C——C (Fig 4C). The closest vertebrate homologs of TSP-21 are TSPAN4, TSPAN9 and CD53 (Figs 4C and S1). These proteins, except for CD53, share the conserved C6a configuration in the EC2 loop as well as conserved transmembrane (TM) domains (Fig 4C). The G109E mutation in *jj60* affects the last residue of TM3 (Fig 4A and 4C), and likely results in the production of a partial loss-of-function TSP-21 protein. The deleted residues of TSP-21 in *jj77* mutants include one of the six highly conserved cysteine residues in EC2 (Fig 4C). *jj77* also contains a missense mutation in a conserved residue in TM4 (V236E, Fig 4), and is therefore likely a strong loss-of-function, or likely null, allele of *tsp-21*. We have therefore used *jj77* for all our subsequent analyses.

### TSP-21 positively modulates the Sma/Mab pathway at the level of the ligand-receptor complex

Because our *sma-9* suppressor screen is highly specific in identifying components of the BMP-like Sma/Mab pathway, we examined *tsp-21(jj77)* mutants for any additional Sma/Mab signaling defects, such as body size, male tail patterning and expression of RAD-SMAD, a Sma/Mab signaling reporter that we previously generated [21]. *tsp-21(jj77)* animals are smaller than wild-type animals (Fig 2B–2E) and exhibited reduced RAD-SMAD reporter expression (Fig 3). Unlike mutants in core members of the Sma/Mab pathway, *tsp-21(jj77)* mutant males can mate and they do not exhibit any significant male tail patterning defects (based on examining 100 sides of *tsp-21(jj77)* male tails). These *tsp-21* phenotypes are very similar to those exhibited by null mutants in two previously identified Sma/Mab pathway modulators, *drag-1* and *unc-40* [21, 22]. Furthermore, like mutants in other members of the Sma/Mab pathway [20–22], *tsp-21(jj77)* mutants exhibited no M lineage defects (Table 3). Finally, *tsp-21(jj77)* mutants showed no dauer defects, and *tsp-21* exhibited no genetic interaction with *daf-1* and *daf-7*, two genes functioning in the TGFβ-like dauer pathway ([12]; S1 Table). Collectively, these phenotypic analyses suggest that TSP-21 positively modulates the BMP-like Sma/Mab pathway, but does not appear to play a role in the TGFβ-like dauer pathway.

The smaller body size of *tsp-21(jj77)* mutants allowed us to use genetic epistasis to determine where in the Sma/Mab pathway TSP-21 functions. We generated double mutants between *tsp-21(jj77)* and null mutations in various Sma/Mab pathway components (Fig 2A) and measured their body sizes. As shown in Fig 2E, *dbl-1(wk70); tsp-21(jj77)* and *sma-3(jj3); tsp-21(jj77)* double mutants were as small as *dbl-1(wk70)* and *sma-3(jj3)* single mutants, respectively. These observations are consistent with TSP-21 functioning in the Sma/Mab pathway in regulating body size. *lon-1(jj67); tsp-21(jj77)* double mutants were as long as *lon-1*(*jj67)* single mutants, while *lon-2(e678) tsp-21(jj77)* double mutants showed intermediate body size between *lon-2(e678)* and *tsp-21(jj77)* single mutants (Fig 2E), suggesting that *tsp-21* is likely to function upstream of *lon-1*, but in parallel to *lon-2*, in the Sma/Mab pathway. *drag-1(jj4); tsp-21(jj77)* and *unc-40(e1430); tsp-21(jj77)* double mutants, or *drag-1(jj4) unc-40(e1430); tsp-21(jj77)* triple mutants were significantly smaller than each respective single mutant (Fig 2E), which is consistent with *tsp-21* functioning in parallel to *drag-1* and *unc-40*. Taken together, these results indicate that TSP-21 functions at the ligand-receptor level to positively modulate Sma/Mab signaling.

### TSP-21 is localized to the plasma membrane and functions in the hypodermis and M lineage, which are Sma/Mab signal-receiving cells

**Fig 5:**
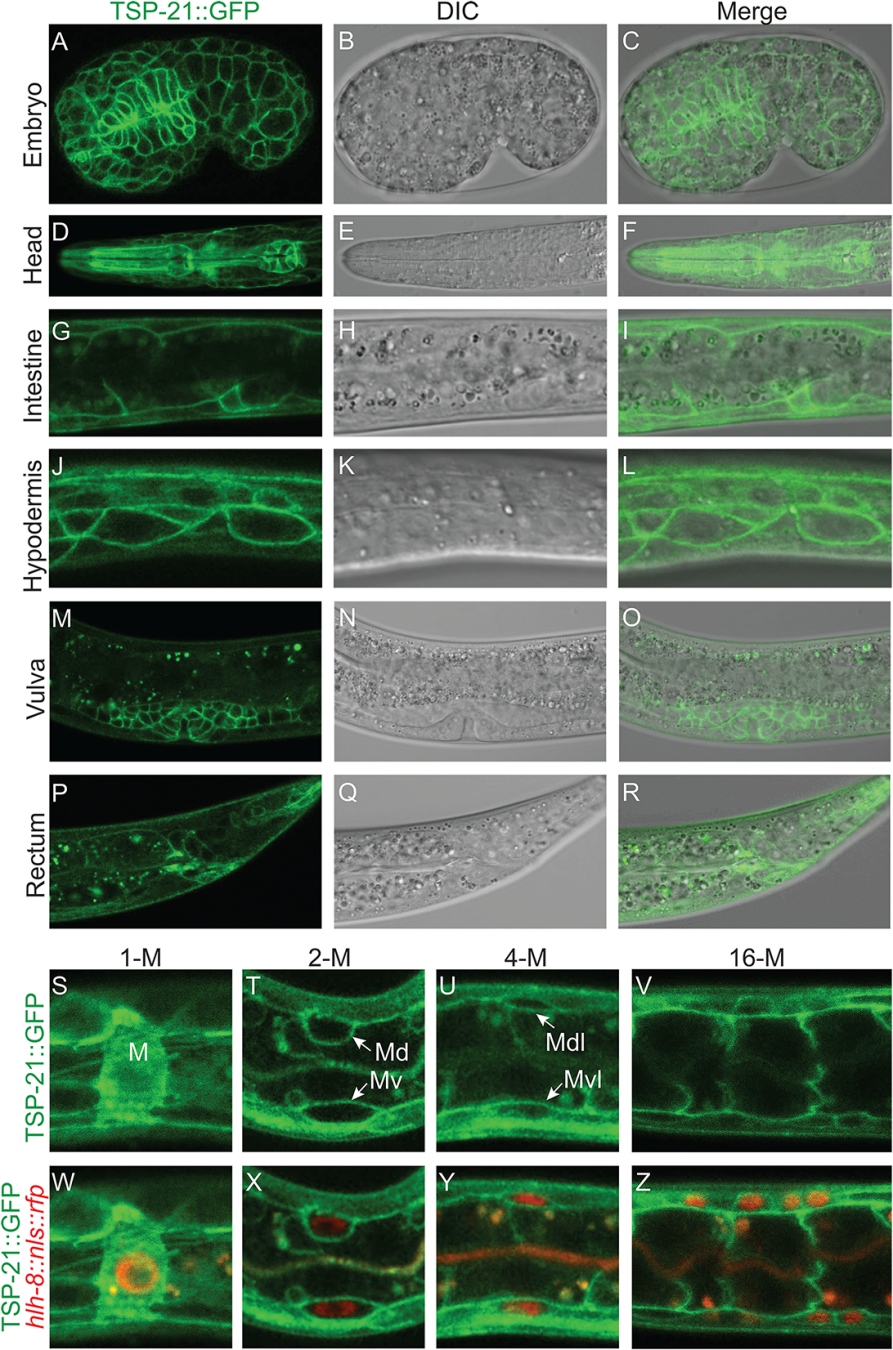
TSP-21 is localized to the plasma membrane of multiple cell types, including known Sma/Mab signal-receiving cells. (A-R) Mid-stage embryo (A-C) or L1 larvae (D-L) or L4 larvae (M-R) showing confocal images of TSP-21::GFP (A, D, G, J, M, P), the corresponding DIC (B, E, H, K, N, Q) and merged images (C, F, I, L, O, R). TSP-21::GFP is localized to the plasma membrane of hypodermal and pharyngeal cells in embryos (A-C), pharyngeal cells (D-F), intestinal cells (G-I) and hypodermal cells (J-L) in L1 larvae, the developing gonad (M-O) and rectal epithelium (P-R) in L4 larvae. (S-Z) L1 larvae expressing both TSP-21::GFP and the M lineage specific reporter *hlh-8p*::*nls*::*rfp*. TSP-21::GFP is present in the M lineage from the 1-M stage through the 16-M stage. Some M lineage cells are out of the focal plane and not shown in panels U-V and Y-Z.

To determine how TSP-21 functions in the Sma/Mab pathway, we examined the expression and localization pattern of TSP-21. We first generated integrated transgenic lines carrying a translational TSP-21::GFP fusion (pJKL1004, see Materials and Methods) that contains the entire *tsp-21* genomic region including 5kb 5’ sequences and 1.7kb 3’ sequences (Fig 4B). This translational fusion rescued the Susm phenotypes of *tsp-21(jj77)* mutants (Table 3). Subsequently we generated the same fusion in the endogenous *tsp-21* locus via CRISPR-Cas9 mediated homologous recombination (see Materials and Methods). Both reporters showed that TSP-21::GFP is plasma membrane-localized and is expressed in a wide variety of somatic cell types, including the pharynx, intestine and hypodermis starting in embryos after the 100 cell stage (Fig 5A–5C at mid-embryogenesis) and peaking in L1 and L2 larvae (Fig 5D–5L). The TSP-21::GFP signal in these tissues decreases in late larval and adult stage animals. TSP-21::GFP is also present at the surface of M lineage cells from the 1-M stage to the 16-M stage (Fig 5S–5Z). In addition to expression in these tissues, the two TSP-21::GFP lines generated via the CRISPR-Cas9 system also showed GFP expression in the somatic gonad and vulva in L2-L4 larvae and adults (Fig 5M–5O), as well as in the rectal epithelium in L4 larvae (Fig 5P–5R). These observations suggest that the enhancer elements for *tsp-21* expression in the somatic gonad, the vulva and the rectal epithelium lie outside of the 10.5kb *tsp-21* genomic region included in pJKL1004.

We noticed that TSP-21::GFP is enriched in the basolateral side of intestinal cells while being absent from their apical sides (Fig 5G–5I). A similar localization pattern has been reported for the type I receptor SMA-6 and the type II receptor DAF-4 [38]. In addition, while TSP-21::GFP in the M lineage cells is primarily plasma membrane localized, there is also a significant intracellular distribution of TSP-21::GFP in the M mesoblast (Fig 5S and 5W). At present, the functional significance of either the asymmetric localization of TSP-21::GFP in intestinal cells or its intracellular localization in the M cell is not clear.

The Sma/Mab pathway is known to function in the hypodermal cells to regulate body size and in the M lineage to regulate M lineage development. We next tested whether TSP-21 functions in these cell types to exert its role in Sma/Mab signaling. Using cell-type-specific promoters to drive *tsp-21* expression, we found that forced expression of *tsp-21* cDNA in hypodermal cells, but not in pharyngeal or intestinal cells, rescued the small body size phenotype of *tsp-21(jj77)* mutants (Table 4). Similarly, forced expression of *tsp-21* cDNA in the M lineage also rescued the Susm phenotype of *tsp-21(jj77)* mutants (Table 3). Thus TSP-21 functions autonomously in the signal-receiving cells to promote Sma/Mab signaling.

**Table 4:** Rescue of the small body size of *tsp-21(jj77)* worms by tissue-specific expression of *tsp-21* cDNA.

*tsp-21* allele	Transgene	Tissue of transgene expression	Relative body length
WT	None	None	1.11 ± 0.006*** (n = 34)
*tsp-21(jj77)*	None	None	1.00 ± 0.007 (n = 30)
*tsp-21(jj77)*	*tsp-21p*::*tsp-21 cDNA*	Hypodermis, intestine, pharynx	1.13 ± 0.010*** (n = 34)
*tsp-21(jj77)*	*rol-6p*::*tsp-21 cDNA*	Hypodermis	1.17 ± 0.006*** (n = 34)
*tsp-21(jj77)*	*elt-3p*::*tsp-21 cDNA*	Hypodermis	1.08 ± 0.008*** (n = 72)
*tsp-21(jj77)*	*myo-2p*::*tsp-21 cDNA*	Pharynx	1.00 ± 0.012 (n = 32)
*tsp-21(jj77)*	*elt-2p*::*tsp-21 cDNA*	Intestine	1.01 ± 0.010 (n = 40)

Body length was normalized to that of *tsp-21(jj77)* mutants. For each genotype, data from two transgenic lines were pooled and averaged. Data shown are mean ± s.e.m.

***p<0.0001, different transgenic worms versus *tsp-21(jj77)* (unpaired two-tailed Student’s *t*-test).

### TSP-12 and TSP-14 function redundantly to promote Sma/Mab signaling

There are 21 tetraspanins in *C*. *elegans*. Our finding that TSP-21 functions in Sma/Mab signaling prompted us to ask whether other tetraspanins might also function in the Sma/Mab pathway. We therefore screened through the remaining 20 tetraspanin *tsp* genes by RNAi injection, testing whether any of them are involved in Sma/Mab signaling using the *sma-9* suppression assay. Only *tsp-12(RNAi)* resulted in a low penetrance (9.4%, n = 767) Susm phenotype (Table 5). We then tested a deletion allele of *tsp-12*, *ok239*, and found that it also exhibited the Susm phenotype (Table 5), suggesting that the tetraspanin TSP-12 also plays a role in modulating Sma/Mab signaling. Dunn and colleagues [28] have previously reported that TSP-12 and TSP-14 function redundantly to promote Notch signaling. We asked whether *tsp-14* and *tsp-12* might also share a redundant role in the Sma/Mab pathway, and found that *tsp-14(RNAi)* enhanced the penetrance of the Susm phenotype of the *tsp-12(ok239)* mutation (Table 5). This effect appears to be specific since *tsp-10(RNAi)* failed to enhance the penetrance of the Susm phenotype of the *tsp-12(ok239)* mutation (Table 5). Thus, TSP-12 and TSP-14 also function redundantly to promote Sma/Mab signaling, in addition to their role in Notch signaling.

**Table 5:** TSP-12 and TSP-14 function redundantly to promote Sma/Mab signaling.

Genotype	% of animals with 1–2 M-CCs (total # of animals examined)
*sma-9(cc604)*	2.2 ± 0.51 (N = 768)
*tsp-12(ok239)*	93.7 ± 1.34 (N = 649)
*tsp-21(RNAi) sma-9(cc604)* ^*a*^	39.6 ± 4.6*** (N = 610)
*tsp-10(RNAi);sma-9(cc604)*	1.7 ± 0.42 (N = 671)
*tsp-12(RNAi);sma-9(cc604)* ^*a*^	9.4 ± 0.83*** (N = 767)
*tsp-14(RNAi) sma-9(cc604)*	1.2 ± 0.55 (N = 728)
*tsp-12(ok239);sma-9(cc604)* ^*a*^	15.2 ± 1.94*** (N = 784)
*tsp-10(RNAi);tsp-12(ok239);sma-9(cc604)*	8.5 ± 1.51 (N = 760)
*tsp-12(RNAi) tsp-12(ok239);sma-9(cc604)*	8.0 ± 1.16 (N = 528)
*tsp-12(ok239); tsp-14(RNAi) sma-9(cc604)* ^*b*^	38.7 ± 3.81*** (N = 438)

*tsp-21(RNAi)* was used as a positive control for the *sma-9* suppression assay.

RNAi of other *tsp* genes (*tsp-1*, *2*, *3*, *4*, *5*, *6*, *7*, *8*, *9*, *10*, *11*, *13*, *16*, *17*, *18*, *19*, *20*) gave similar results as *tsp-10(RNAi)*, while *tsp-15(RNAi)* resulted in embryonic/larval lethality, consistent with previous reports on *tsp-15* function [105].

***p<0.0001 (unpaired two-tailed Student’s *t*-test)

^a^ *tsp; sma-9(cc604)* double mutants vs. *sma-9(cc604)* single mutants.

^b^ *tsp-12(ok239); tsp-14(RNAi) sma-9(cc604)* mutants vs. *tsp-12(ok239); sma-9(cc604)* mutants.

### TSP-21 positively modulates LIN-12/Notch signaling in the M lineage

The dual functions of TSP-12 and TSP-14 in both the Notch and the Sma/Mab signaling pathways prompted us to examine whether TSP-21 also functions in the Notch signaling pathway. The LIN-12/Notch signaling pathway is known to function in the M lineage to promote the ventral fate: loss of LIN-12/Notch function results in a ventral-to-dorsal fate transformation in the M lineage, namely the loss of M-derived SMs and the gain of M-derived CCs ([39, 40]; S2 Fig). *tsp-21(jj77)* single mutants exhibit no M lineage defects. We therefore examined whether *tsp-21(jj77)* could enhance the M lineage defect of the *lin-12* temperature sensitive, partial loss-of-function allele *lin-12(n676n930ts)* by scoring the number of M-derived CCs. As shown in Table 6, *tsp-21(jj77)* significantly enhanced the M lineage defect of *lin-12(n676n930ts)* at both 20°C and 22°C, suggesting that TSP-21 functions to promote LIN-12/Notch signaling in the M lineage. However, *tsp-21(jj77)* failed to enhance the sterility and embryonic lethality of *bn18ts*, a mutation in the second Notch receptor gene in *C*. *elegans*, *glp-1* [41]. The lack of genetic interaction between *tsp-21(jj77)* and *glp-1(bn18)* is consistent with the absence of TSP-21::GFP expression in the germline and early embryo, as described above.

**Table 6:** TSP-21 positively modulates LIN-12/Notch signaling in the M lineage.

Genotype	1–2 M-CCs	3–4 M-CCs	N
Wild-type [20°C]	100%	0%	>100
*tsp-21(jj77)* [20°C] and [22°C]	100%	0%	>100
*lin-12(n676n930ts)* [20°C]	99.0%	1.0%	279
*lin-12(n676n930ts); tsp-21(jj77)* [20°C]	93.0%	7.0%***	356
*lin-12(n676n930ts)* [22°C]	86.0%	14.0%	326
*lin-12(n676n930ts); tsp-21(jj77)* [22°C]	64.4%	35.6%***	623

***p<0.001, *lin-12(n676n930ts); tsp-21(jj77)* versus *lin-12(n676n930ts)* worms (unpaired two-tailed Student’s *t*-test).

### TSP-21 can form a complex with TSP-12 and TSP-14

**Fig 6:**
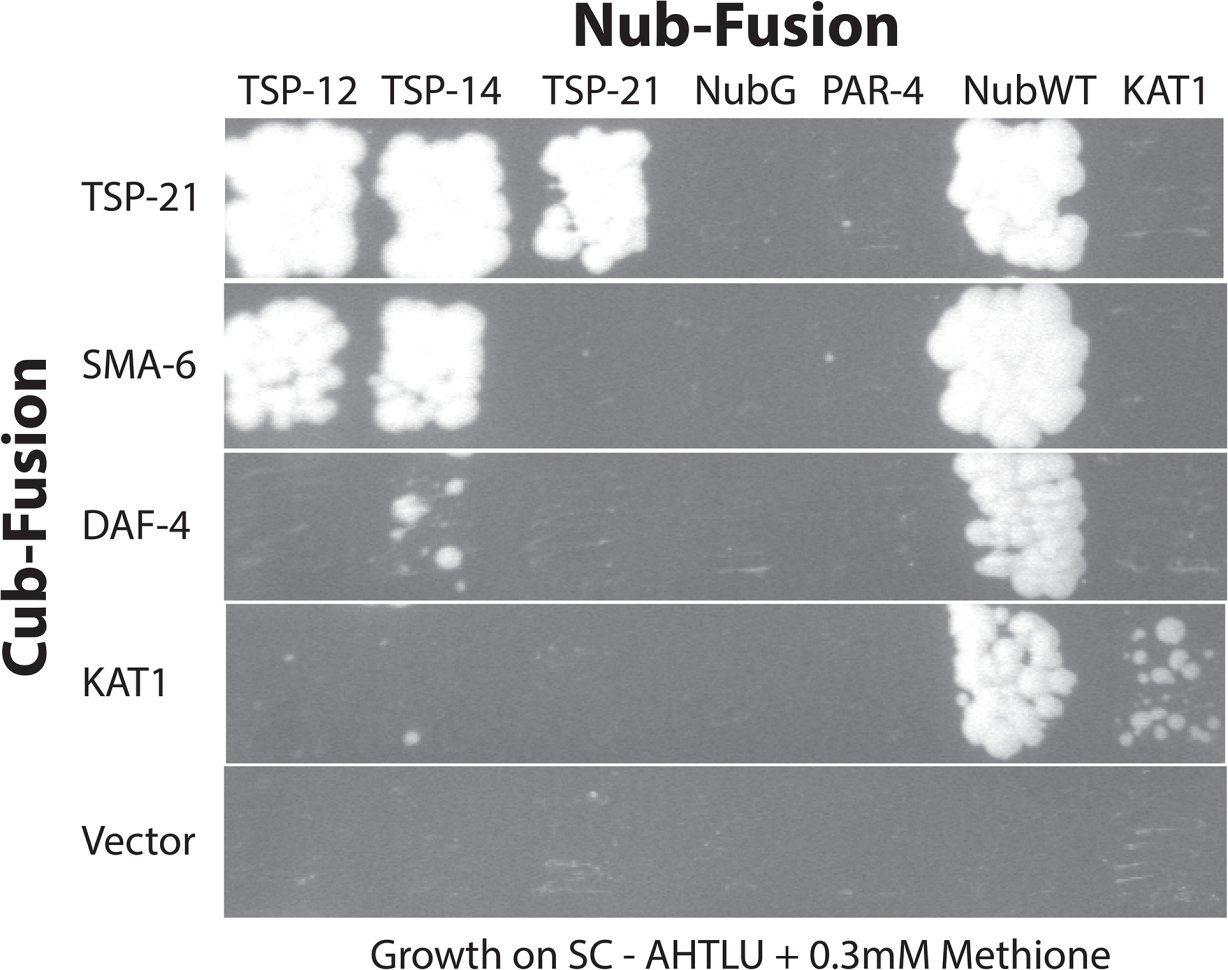
Interactions among TSP-12, TSP-14, TSP-21 and the receptors SMA-6 and DAF-4. Diploid yeast cells expressing specific Cub- and Nub- fusion proteins were grown in SC-Ade,-His, Trp,-Leu,-Ura (SC-AHTLU) plates supplemented with 0.3mM of methionine. Cub and NubG were each used as negative controls. NubWT was used as a positive control to indicate expression of each Cub-PLV fusion protein. The potassium channel KAT1 was used as a control for specificity. KAT1 can interact with KAT1, but not with any of the proteins tested here.

Tetraspanins often associate with each other and with other membrane or membrane-associated proteins to organize membranes into tetraspanin-enriched microdomains [24–26]. Our finding that in addition to TSP-21, TSP-12 and TSP-14 also function in promoting Sma/Mab signaling suggested that these tetraspanins might interact with each other. We tested this hypothesis by using the mating-based split-ubiquitin system (mbSUS, [42]) in budding yeast. The mbSUS is based on the observation that a full-length ubiquitin can be reconstituted when the N-terminal ubiquitin domain (Nub) and the C-terminal ubiquitin domain (Cub) are brought into close proximity [43, 44]. This system can be used to identify potential interactions between full-length membrane proteins or between a membrane protein and a soluble protein: a mutant form of Nub, NubG, that has reduced affinity for Cub, can only reconstitute with Cub via two interacting proteins. The reconstituted ubiquitin will direct ubiquitin-specific proteases to liberate PLV (protein A, LexA and VP16) from Cub, which then enters the nucleus and activates transcription of reporter genes. We generated TSP-Cub fusions and Nub-TSP or TSP-Nub fusions (see Materials and Methods, and S2 Table for a list of the plasmids generated), and tested pairwise interactions among the three tetraspanins, as well as interactions between these tetraspanins and the type I and type II receptors SMA-6 and DAF-4, respectively. Results from these experiments are summarized in Fig 6. TSP-12-Cub appeared to auto-activate reporter expression, while the TSP-14-Cub was not detectable on western blots (see Materials and Methods). For the remaining three Cub fusions (TSP-21, SMA-6 and DAF-4), we found that TSP-21 can associate with itself, as well as with TSP-12 and TSP-14 (Fig 6). In addition, SMA-6 can associate with both TSP-12 and TSP-14, but not TSP-21. We also detected a very weak interaction between DAF-4 and TSP-14 (Fig 6). The use of multiple positive and negative controls in these experiments (see Materials and Methods, and Fig 6) indicated that the observed interactions are highly specific. For example, TSP-21 did not show any interaction with the *C*. *elegans* LKB homolog PAR-4 ([45]; Fig 6), or the plant potassium channel KAT1 ([42]; Fig 6), or with the *C*. *elegans* ABC transporter HMT-1 [46]. Except for the weak DAF-4-TSP-14 interaction, the other observed interactions all appeared to be particularly strong, as yeast growth on SC-Trp,-Leu,-Ade,-His,-Ura,-Met plates supplemented with 0.3mM of methionine was detectable only 2 days after streaking the mated yeast. Thus, TSP-21 can form both homo-oligomers and heteromeric complexes with TSP-12 and TSP-14. These findings are consistent with our genetic evidence that all three tetraspanins function to promote Sma/Mab signaling. The strong interactions between SMA-6 and TSP-12 and TSP-14 suggest that these tetraspanins might function by directly recruiting the receptor molecules to specific membrane microdomains.

### Glycosphingolipids are required for promoting Sma/Mab signaling

Tetraspanin-enriched microdomains are also enriched in cholesterol and glycosphingolipids [24–26]. We therefore tested whether cholesterol and/or glycosphingolipids are required for Sma/Mab signal transduction. Our results suggest that Sma/Mab activity is influenced by glycosphingolipids but not cholesterol.

*C*. *elegans* worm survival requires exogenous cholesterol [47, 48]. In the lab, worms are normally fed with *E*. *coli* bacteria on agar plates supplemented with 5μg/mL cholesterol [49]. Using a method that can lead to nearly complete cholesterol depletion ([50, 51] and see Materials and Methods), we grew L1 or L4 worms on cholesterol-depleted plates and scored their phenotypes or their progeny’s phenotype, respectively, at the adult stage. We found no suppression of the M lineage phenotype when *sma-9(cc604)* worms were grown on cholesterol-depleted media, even though the worms were sterile, a known phenotype resulting from cholesterol depletion [47, 48]. Thus cholesterol does not seem to be essential for Sma/Mab signaling.

To determine the requirement of glycosphingolipids in Sma/Mab signaling, we generated double mutants between *sma-9(cc604)* and mutations that reduce or eliminate the activity of enzymes involved in glycosphingolipid biosynthesis [52], S3 Fig), and examined their Susm phenotype. As shown in Table 7, mutations in *cgt-3* and *bre-5* partially suppressed the *sma-9* M lineage phenotype. *cgt-3* encodes the ceramide glucosyltransferase that converts ceramide to glucosylceramide, a precursor of complex glycosphingolipids [53, 54]. Previous work has shown that CGT-3 is the major enzyme among the three worm CGT proteins [54]. We found that a deletion allele of *cgt-3*, *ok2877*, which deletes most of the coding exons of *cgt-3*, resulted in a late L1 or early L2 larval arrest and a partial suppression of the *sma-9(cc604)* M lineage defects (Table 7). *cgt-3(ok2877)* mutants exhibited additional defects in Sma/Mab signaling: the relative fluorescence intensity of the RAD-SMAD reporter in *cgt-3(ok2877)* mutants is only 58% of that in stage-matched wild-type animals (see Materials and Methods) and *cgt-3(ok2877)* mutants exhibited a smaller body size compared to stage-matched wild-type control animals (72% of wild-type body length, n = 23). We also observed a low penetrance of the Susm phenotype in *ye17*, an allele of *bre-5* that encodes a β-1,3-galactosyltransferease involved in glycosphingolipid biosynthesis (Table 7, [55, 56]). Taken together, our data suggest that glycosphingolipids are required for Sma/Mab signaling. The lack of a Susm phenotype for the other mutations affecting glycosphingolipid biosynthesis (Table 7) may be because many of them are partial loss-of-function alleles, since null mutations in many of these genes result in lethality [52]. Alternatively, proper Sma/Mab signaling may require specific type(s) of glycosphingolipids.

**Table 7:** Mutations in enzymes critical for glycosphigolipid (GSL) biosynthesis are defective in Sma/Mab signaling.

Genotype (allele)	Protein product of mutated gene^a^	% of animals with 1–2 M-CCs (# of animals examined)
*sma-9(cc604)*	—	3.00% (n = 100)
pod-2(ye60);sma-9(cc604)**	acetyl-CoA carboxylase	3.00% (n = 198)
*fat-2(wa17);sma-9(cc604)*	δ-12 fatty acyl desaturase	0.01% (n = 580)
*fat-3(wa22);sma-9(cc604)*	δ-6 fatty acyl desaturase	0.01% (n = 512)
*fat-6(tm331);fat-7(wa36);sma-9(cc604)*	δ-9 fatty acyl desaturase	2.12% (n = 848)
*sptl-1(ok1693);sma-9(cc604)*	serine palmitoyl transferase	0.00% (n = 264)
*cgt-1(ok1045);sma-9(cc604)*	ceramide glucosyltransferase-1	0.24% (n = 410)
cgt-3(ok2877);sma-9(cc604))*	**ceramide glucosyltransferase-3**	**46.47% (n = 420)**
*cgt-3(ok2877);sma-9(cc604); jjEx[hlh-8p*::cgt-3]*,^*b*^		32.17%*** (n = 325)
*bre-1(ye4);sma-9(cc604)*	GDP-mannose 4, 6-dehydratase	0.92% (n = 326)
*bre-2(ye31);sma-9(cc604)*	β1,3-glycosyltransferase	0.90% (n = 443)
*bre-3(ye26);sma-9(cc604)*	β3-mannosyltransferase	5.00% (n = 505)
*bre-3(ye28);sma-9(cc604)*	β3-mannosyltransferase	3.00% (n = 231)
*bre-4(ok3167);sma-9(cc604)*	β-4-N-acetylgalactosaminyltransferase	0.00% (n = 218)
***bre-5(ye17);sma-9(cc604)***	**β-3-N-acetylglucosaminyltransferase**	**13.4% (n = 433)**

Animals were growing at 20°C and scored at the L4 stage except for cgt-3(ok2877)* animals, which were arrested at late L1/early L2, and for the cold-sensitive pod-2(ye60)** animals which were growing at 15°C during postembryonic development.

***p<0.0001, *cgt-3(ok2877); jjEx[hlh-8p*::*cgt-3]* versus *cgt-3(ok2877)* worms (unpaired two-tailed Student’s *t*-test).

^a^ The *sma-9* gene product is not listed in this column.

^b^ Data generated by averaging the results from three independent transgenic lines.

*cgt-3* is widely expressed in multiple cell types in *C*. *elegans* [53, 54]. We tested whether *cgt-3*, and therefore glycosphingolipids, are required in the signal-receiving cells for proper Sma/Mab signaling. Expression of *cgt-3* in the M lineage using the *hlh-8* promoter partially, but significantly, rescued the Susm phenotype of *cgt-3(ok2877)* mutants (Table 7), suggesting that proper Sma/Mab signaling requires glycosphingolipids in the signal-receiving cells. The lack of complete rescue suggests that glycosphingolipids are also required outside of the signal-receiving cells to promote Sma/Mab signaling.

During the course of our study, we observed that both *cgt-3(ok2877)* and *bre-5(ye17)* single mutants exhibited a low penetrance M lineage phenotype like that of a *lin-12(lf)* mutant: extra M-derived CCs due to the fate transformation of M-derived SMs to CCs ([39, 40]; Table 8 and S2 Fig). We further found that *cgt-3(ok2877)* enhanced the penetrance of the M lineage defects of a hypomorphic *lin-12* temperature sensitive allele, *n676n930*, at a semi-permissive temperature (Table 8). These observations are consistent with previous findings by Katic and colleagues [57] showing that enzymes required for glycosphingolipid biosynthesis, such as BRE-5, are required for promoting LIN-12/Notch signaling. The requirement of glycosphingolipids in LIN-12/Notch signaling appears to be distinct from their requirement in Sma/Mab signaling. Mutations in the Sma/Mab pathway fully restore the *sma-9(0)* M lineage phenotype back to that of wild-type animals ([20–22, 40]; S2 Fig). However, *lin-12(0); sma-9(0)* double mutants exhibit a reversal of the M lineage dorsoventral polarity, so that the double mutants have 2 SMs born on the dorsal side and 2 M-derived CCs located on the ventral side ([39, 40]; S2 Fig). Careful examination of the position of the M-derived CCs in *cgt-3(ok2877);sma-9(cc604)* and *bre-5(ye17);sma-9(cc604)* mutants showed that a majority of the double mutant animals have their M-derived CCs located on the dorsal side (S3 Table), indicating a suppression rather than a reversal of polarity. Taken together, our results support the notion that glycosphingolipids are required for both LIN-12/Notch and Sma/Mab signaling.

**Table 8:** *cgt-3* and *bre-5* mutants exhibit defects in LIN-12/Notch signaling in the M lineage.

Genotype	0 M-CCs	1–2 M-CCs	3–4 M-CCs	N
Wild-type [20°C]	0%	100%	0%	>100
*bre-5 (ye17)* [20°C]	0%	92.50%	7.50%	106
*cgt-3(ok2877)* [22°C]	0%	84.50%	15.50%	97
*lin-12(n676n930ts)* [22°C]	0%	91.96%	8.04%	87
*cgt-3(ok2877); lin-12(n676n930ts)* [22°C]	0%	66.10%	33.90%	109

## Discussion

### Tetraspanins play important roles in promoting BMP signaling

In this study, we identified TSP-21, a C6a class tetraspanin, as a key factor promoting the BMP-like Sma/Mab signaling in *C*. *elegans*. *tsp-21* mutants exhibit small body size and Susm phenotypes similar to that shown by mutants in core Sma/Mab pathway components. The TSP-21 protein is localized to the plasma membrane, and *tsp-21* is expressed and functions in the signal-receiving cells at the ligand-receptor level to promote Sma/Mab signaling. We found that among the remaining 20 *C*. *elegans* tetraspanins, TSP-12 and TSP-14 function redundantly to also promote Sma/Mab signaling. How do these three tetraspanins function to promote Sma/Mab signaling? We envision two possible, non-mutually exclusive, scenarios.

**Fig 7:**
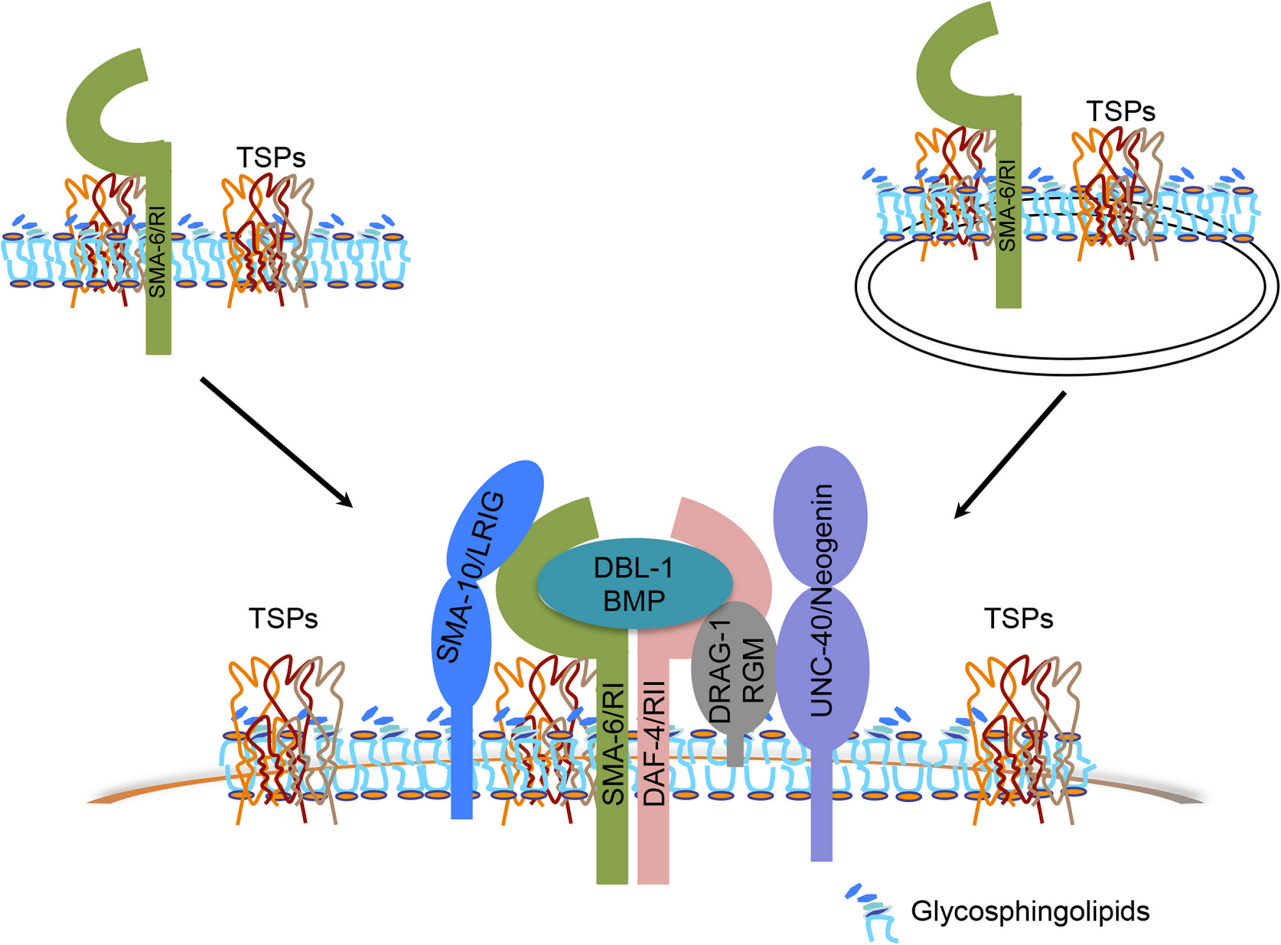
Models of how tetraspanin proteins function to promote BMP signaling. Tetraspanins TSP-21, TSP-12 and TSP-14 (collectively TSPs) interact with each other to organize membranes into microdomains that are enriched in glycosphingolipids (GSLs). Via interacting with the type I receptor SMA-6, the tetraspanins may recruit the receptors or the ligand-receptor complex (DBL-1/BMP, SMA-6/RI, DAF-4/RII) and their associated modulators (DRAG-1/RGM, UNC-40/neogenin and SMA-10/LRIG) to these microdomains, resulting in enhanced BMP signaling. Alternatively, but not mutually exclusively, these tetraspanins may function to regulate the trafficking of their associated receptor SMA-6 and possibly other components in the Sma/Mab pathway to promote BMP signaling.

In the first scenario, the three tetraspanins might promote clustering of the receptor complexes or the ligand-receptor complexes to modulate Sma/Mab signaling. Tetraspanins are known to homo- and hetero-oligomerize to organize membranes into tetraspanin-enriched microdomains, which are also enriched in tetraspanin-associated proteins [24–26]. Previous work has shown that in mouse, TSPAN12 promotes Norrin/β-catenin signaling by enhancing clustering of the Norrin receptor FZD4 [58, 59]. In particular, TSPAN12 and Norrin can each enhance FZD4 clustering but work together cooperatively to further increase the clustering of the ligand-receptor complex to promote Norrin/β-catenin signaling [58]. We have shown that *C*. *elegans* TSP-12, -14 and -21 can interact with each other in yeast and that both TSP-12 and TSP-14 can interact with the type I receptor SMA-6. In addition, we found that glycosphingolipids, which are enriched in tetraspanin-enriched microdomains, are also required for proper Sma/Mab signaling. These findings suggest that TSP-21, TSP-12 and TSP-14 may function by recruiting the receptor complex, or the ligand-receptor complex, to glycosphingolipid-enriched membrane microdomains containing TSP-21-TSP-12-TSP-14, thereby increasing the local concentration of the receptors, or the ligand-receptor complexes, to promote Sma/Mab signaling (Fig 7). Supporting this model, SMA-6, DAF-4 and TSP-21 are all localized to the basolateral membranes of the polarized intestinal cells ([38]; this work). We envision that several previously identified positive modulators of the Sma/Mab pathway, including DRAG-1/RGM, UNC-40/neogenin, and SMA-10/LRIG, might be localized in these microdomains as well, as all three proteins are plasma membrane-localized, are expressed and function in the signal-receiving cells, and interact with the ligand and the receptors (for DRAG-1), or the receptors (for SMA-10), or with each other (for DRAG-1 and UNC-40) [21, 22, 34]. Further biochemical and cell biological experiments are needed to determine the presence and subcellular localization of TSP-21-TSP-12-TSP-14-containing membrane microdomains, whether the Sma/Mab pathway receptors and modulators are indeed localized to these microdomains, and what other factors are also present there.

Alternatively, but not mutually exclusively, the three tetraspanins might be involved in the trafficking of essential Sma/Mab pathway components. Tetraspanins have been found to be present in the plasma membrane or various types of intracellular membranous organelles, and multiple tetraspanins are known to regulate the processing and trafficking of associated proteins [60]. In *C*. *elegans*, TSP-12 and TSP-14 have previously been shown to function redundantly in promoting Notch signaling [28]. Their *Drosophila* and mammalian homologs, the TspanC8 tetraspanins, interact with the ADAM (a disintegrin and metalloprotease) protease ADAM10 to promote its maturation and trafficking to the cell surface, which in turn promotes Notch signaling [61–63]. TSP-12 and TSP-14 may function in a similar manner in promoting Sma/Mab signaling. Since both TSP-12 and TSP-14 can bind to the type I receptor SMA-6 in yeast, they may promote Sma/Mab signaling by regulating the trafficking of SMA-6 (Fig 7), and/or other players in the Sma/Mab pathway. Further work is needed to test this hypothesis. Since the role of TspanC8 tetraspanins in promoting Notch signaling is evolutionarily conserved [61–63], it will be interesting to determine whether the role of TspanC8 tetraspanins in modulating BMP signaling is also evolutionarily conserved, and whether these tetraspanins function in a similar manner in promoting both BMP and Notch signaling.

Using *C*. *elegans* as a model, Gleason and colleagues recently showed that the type I receptor SMA-6 and the type II receptor DAF-4 utilize distinct mechanisms for their intracellular recycling, providing physiological evidence supporting the roles of endocytosis and intracellular trafficking in regulating BMP signaling [38]. In light of the roles of multiple tetraspanins in regulating the processing and trafficking of associated proteins [60], our findings, together with that of Gleason and colleagues [38], highlight the usefulness of *C*. *elegans* as a model system in identifying cell biological mechanisms that regulate BMP signaling.

### Possible links between tetraspanins, cancer and TGFβ or BMP signaling

The family of tetraspanin proteins is large: there are 21 tetraspanins in *C*. *elegans* and 33 tetraspanins in humans. Recent studies have implicated tetraspanins in multiple diseases and physiological processes in humans [60]. In particular, several tetraspanins, such as CD151 [64], TSPAN12 [65], and TSPAN8 [66], among others, have been implicated in cancer initiation, progression and metastasis in mammals. These and other tetraspanins have emerged as diagnostic and prognostic markers, and possible therapeutic targets, for tumor progression (for reviews, see [27, 67]). However, the mechanism by which the mis-regulation of these tetraspanins contributes to cancer is not fully understood [27, 67]. It is well known that mis-regulation of TGFβ signaling contributes to cancer initiation and progression [6, 68]. CD151 is the only tetraspanin whose role in cancer has been directly linked to altered TGFβ signaling [69]. Sadej and colleagues showed that CD151 is required for TGFβ1-induced proliferation and scattering of breast cancer cell line MDA-MB-231 through regulating TGFβ-induced p38 phosphorylation, rather than canonical TGFβ-induced Smad phosphorylation. Furthermore, this function of CD151 in TGFβ signaling requires its interaction with the integrins [69]. How CD151-integrin interaction regulates TGFβ-induced p38 phosphorylation is not clear. Recently a study on the tetraspanin-interacting protein EWI-2 indirectly implicates two other tetraspanins, CD9 and CD81, in regulating TGFβ signaling in melanoma growth and metastasis [70]. But the detailed mechanism on how these two tetraspanins regulate TGFβ signaling is not known.

We have provided a direct in vivo link between BMP signaling and three tetraspanins, TSP-21, TSP-12 and TSP-14, in living animals using *C*. *elegans* as a model. Our genetic epistasis results showed that TSP-21 acts through SMA-3, one of the R-Smads in the canonical BMP-like Sma/Mab signaling pathway (Fig 2E). Due to the embryonic arrest of null mutants in the *C*. *elegans* integrin genes, we could not determine whether the function of TSP-21 in Sma/Mab signaling is dependent on integrins. We have found that strong-loss-of function mutations in one of the two *C*. *elegans* genes encoding the α subunit of integrin, *ina-1(gm39)* and *ina-1(gm144)* [71], did not exhibit any Susm phenotype (n = 53 for *gm39*, and n = 109 for *gm144*). But we cannot rule out the possibility that in these mutants residual *ina-1* function or function of *pat-2*, another gene encoding the α subunit of integrin [72] is sufficient to mediate Sma/Mab signaling.

TSP-21 is orthologous to human TSPAN4, TSPAN9 and CD53, but is much more distantly related to CD151 (whose *C*. *elegans* ortholog is TSP-17; S1 Fig). It is therefore possible that the differences between CD151 and TSP-21 in regulating TGFβ signaling are due to intrinsic biochemical differences between the two types of proteins. Alternatively, since TSP-21 regulates a BMP-like Sma/Mab signaling pathway, it is likely that tetraspanins can regulate both TGFβ signaling and BMP signaling, but via distinct downstream effectors. Interestingly, each of the three human orthologs of TSP-21 (TSPAN4, TSPAN9 and CD53), as well as two out of the six human orthologs of TSP-12 and TSP-14 (TSPAN10 and TSPAN33), are expressed at elevated levels in certain cancer cell lines or tumors [73–75] In addition, one human ortholog of TSP-12 and TSP-14 (TSPAN14) is genetically altered in non-small-cell lung cancer [76]. However, the functional significance of their overexpression or mutation in human cancers is not fully understood. We propose that the involvement of these tetraspanins in cancer may be partially due to their role in modulating the activity of TGFβ and/or BMP signaling.

### Screening for *sma-9* suppressor is a highly specific and sensitive method for identifying key players in the BMP-like Sma/Mab pathway in *C*. *elegans*

Previous genetic studies in *C*. *elegans* have led to the identification of key players in BMP signaling (for example, [16, 17]). A screen based on the body size phenotype has also been fruitful in identifying factors involved in modulating Sma/Mab signaling, such as SMA-10/LRIG [32] and LON-2/glypican [34, 77]. Potential modulators of the Sma/Mab pathway may also exist among a collection of mutants with a small body size phenotype [78]. However, it may be difficult to identify the genes for which mutations produce only a subtle effect on body size, such as *tsp-21(jj77)*. Furthermore, since genes not functioning in the Sma/Mab pathway also regulate body size (for example, [30–32, 79]), not all mutations affecting body size will identify factors specifically functioning in the Sma/Mab pathway. The *sma-9* suppressor screen appears to be a highly specific and sensitive means to identify new components of the Sma/Mab pathway: (1) Mutations in all (except for *crm-1*, see Table 1) previously identified Sma/Mab pathway members suppress the *sma-9* M lineage phenotype (Table 1 and Table 2). In general, partial loss-of-function alleles for a given gene exhibited lower penetrance of the Susm phenotype compared to putative null alleles (Table 2), demonstrating that the suppression of the *sma-9* M lineage phenotype is highly sensitive to altered levels of Sma/Mab signaling. (2) Mutations in other signaling pathways, such as the dauer pathway or the Wnt pathway, or mutations that exclusively affect body size without affecting Sma/Mab signaling, do not suppress the M lineage phenotype of the *sma-9* mutant ([20]; Table 1). (3) Using this screen, we have identified three evolutionarily conserved modulators of the Sma/Mab pathway, DRAG-1/RGM [21], UNC-40/neogenin/DCC [22], and TSP-21/TSPAN4,9 (this study). Additional modulators of this pathway probably exist, as our screen has only recovered single alleles of several genes known to function in Sma/Mab signaling and is, therefore, unlikely to be genetically saturated (Table 2).

In summary, we have developed a highly specific and sensitive way to identify new modulators of the BMP pathway in *C*. *elegans*. This genetic approach has confirmed known regulators and identified novel players. Because of the high degree of conservation of the BMP pathway, the factors that we identify in our screen and the mode of their action that we decipher in *C*. *elegans* will be broadly relevant in understanding modulation of BMP signaling in other metazoans, including humans.

## Materials and Methods

### *C*. *elegans* strains

Strains were grown using standard culture conditions, as described by Brenner [49]. Analyses were performed at 20°C, unless otherwise noted. Cholesterol depletion conditions were following those described in Merris et al. [80] by replacing agar with agarose, and by growing bacteria OP50 and *C*. *elegans* worms on defined media, which contains 3.5mM Tris.HCl, 2mM Tris, 34mM NaCl, and 3.1g/L of ether-extracted peptone. Eggs or L4 hermaphrodite animals were placed on cholesterol-depleted plates and the resulting adult animals were scored for M lineage phenotypes.

The following mutations and integrated transgenes were used: Linkage group I (LG I): *drag-1(jj4)*, *arIs37(secreted CC*::*gfp)*, *bre-4(ok3167)*, *bre-5(ye17);* LG II: *sma-6(e1482)*, *pod-2(ye60)*, *cgt-3(ok2877)/mIn1[mIs14 dpy-10(e128)]*, *jjIs2437[CXTim50*.19[pCXT51(5*RLR::*deleted pes-10p*::*gfp) + LiuFD61(mec-7p*::*rfp)]*, *sptl-1(ok1693)*; LG III: *daf-4(m63)*, *daf-7(m62)*, *sma-2(e502)*, *sma-3(e491)*, *sma-4(e729)*, *lon-1(e185)*, *cup-5(ar465)*, *ina-1(gm144)*, *ina-1(gm39)*, *bre-2(ye31)*, *bre-3(ye26)*, *bre-3(ye28)*, *lin-12(n676n930ts)*, *hT2[qIs48]*, *ccIs4438[intrinsic CC*::*gfp];* LG IV: *daf-1(m40)*, *daf-1(m213)*, *fat-2(wa17)*, *fat-3(wa22)*, *fat-6(tm331)*, *tsp-12(ok239)*, *nT1[qIs51]*; LG V: *dbl-1(wk70)*, *fat-7(wa36)*, *sma-10(wk89)*, *sma-10(ok2224)*, *sma-1(ru18)*, *lon-3(ct417)*, *crm-1(tm2218)*, *him-5(e1467)*, *bre-1(ye4)*, *bre-5(ye17)*, *cgt-1(ok1045)*, *acs-1(gk3066)V/nT1[qIs51]IV;V;* LG X: *lon-2(e678)*, *tsp-21(tm6269)*, *sma-9(cc604)*, *jjIs2433[RAD-SMAD*: *CXTim50*.1[pCXT51(5*RLR::*deleted pes-10p*::*gfp) + LiuFD61(mec-7p*::*rfp)*]].

*tsp-21* and *sma-9* are located 0.79 map unit apart from each other on the X chromosome. We therefore separated the *tsp-21(jj77)* mutation from *sma-9(cc604)* via recombination. Specifically, progeny from *tsp-21(jj77) sma-9(cc604)/+ +* heterozygous parents were scored for the number of CCs. Animals with 6 CCs (*jj77 cc604/+ +* or *+ +/+ +* or *jj77 cc604/jj77 +*) were genotyped for *jj77* homozygosity by PCR. *jj77 cc604/jj77 +* animals were selected and their progeny were further genotyped by sequencing the *sma-9* gene in order to obtain *jj77 +/jj77 +* animals. Four independent recombinants were obtained, #570, #778, #898 and #954. Each recombinant was then outcrossed with N2 three more times before further phenotypic analysis. All four recombinants behaved similarly regarding body size, RAD-SMAD and male tail patterning phenotypes.

The *lon-2(e678) tsp-21(jj77)* double mutant was generated from a *lon-2(e678) egl-15(n484)/tsp-21(jj77)* heterozygous worms by identifying Lon-non-Egl recombinants, and scoring for the presence of the *tsp-21(jj77)* and the *lon-2(e678)* mutations by PCR genotyping.

### RNAi

*let-381(RNAi)* was performed via feeding following the protocol described in [33]. Other RNAi experiments were performed by injection. In general, gene specific fragments were amplified using RNAi clones from the Ahringer library [81] or the Vidal library [82], or using N2 genomic DNA as template. dsRNAs were generated using the T7 Ribomax RNA Production System (Promega) and injected into gravid adult hermaphrodite animals of specific genotypes carrying *CC*::*gfp*. The resulting progeny were scored at the adult stage for the number of CCs.

### Isolation and characterization of *sma-9* suppressor mutations

*arIs37(secreted CC*::*gfp)* I*; cup-5(ar465)* III*; sma-9(cc604)* X animals lacking M-derived coelomocytes (having a total of 4 CCs) were treated with 50 mM ethyl methanesulfonate (EMS). Individual F1 animals were picked to 3F1s per plate and their combined F2 progeny were screened for the restoration of M lineage-derived coelomocytes (having a total of 5–6 CCs) by direct visual examination using a fluorescence stereomicroscope. Plates that segregated 5–25% of animals with 6 CCs were kept for further analysis, including determining whether the mutations bred true, the degree of suppression for each suppressor mutation when homozygous and whether the mutations are dominant or recessive.

By screening through 5,300 haploid genomes using the above method, we isolated 37 true-breeding *sma-9* suppressors, named *susm* (suppressor of *sm* *a-9*) mutations (*jj49-jj85*, Table 2). Four of these, *jj68*, *jj80*, *jj81* and *jj84* showed a relatively low degree of suppression (near 30%, Table 2), and were not further characterized in this work. *jj58* might be a dominant mutation and was not further analyzed. All of the remaining *susm* alleles appear to be recessive, single locus mutations, although some suppressors exhibited partial dominance in their Susm phenotype (Tables 1 and 2). The suppressor mutations were then mapped to chromosome X or chromosome III based on their linkage to *sma-9(cc604)* X or to *cup-5(ar465)* III. Further complementation tests were carried out between each suppressor mutation and mutations in each known members of the Sma/Mab pathway, and between different suppressor mutations that did not affect known genes in the Sma/Mab pathway.

LW0214, which has *arIs37(secreted CC*::*gfp)* I and *sma-9(cc604)* X introgressed into the CB4856 Hawaiian strain by 6x backcrossing, was used for mapping the *sma-9* suppressors via snip-SNP mapping [83] and whole genome sequencing (WGS) [84]. LW0214 was tested using a panel of SNP markers and subsequently by WGS, and found to contain CB4856 SNPs for all six chromosomes except for the following regions that still contain N2 SNP markers: chromosome I—from the left end to -12 and from +24 to the right end; chromosome II—from the left end to -18; and chromosome X—between +1.73 and +11. Snip-SNP markers used were described in Wicks et al. [83] and Davis et al. [85].

For the *sma-9* suppressor mutations that appeared to affect known genes in the Sma/Mab pathway, either by complementation tests, or by whole genome sequencing (WGS, see below), their molecular lesions were identified by sequencing PCR products spanning the entire genomic regions of the corresponding genes, which include *dbl-1*, *daf-4*, *sma-6*, *sma-2*, *sma-3*, *sma-4*, *lon-1*, *sma-10 and unc-40*. For *jj69* that contains a single base pair change in the upstream regulatory region of *sma-6*, a plasmid pJKL1060, which contains 3kb of upstream sequences, the genomic coding region and 2kb of downstream sequences of *sma-6*, was used to rescue the Susm phenotype of *jj69*.

### Whole genome sequencing (WGS) and data analysis

Direct WGS of the homozygous suppressor mutant DNA was performed for some *sma-9* suppressors. For others, the suppressors were simultaneously mapped and identified using the SNP-WGS method of Doitsidou *et al*. [84]. For the SNP-WGS method, each *sma-9; suppressor* mutant was crossed with LW0214, which has *sma-9* introgressed into the polymorphic Hawaiian strain CB4856 (described above). Between 36 and 59 F2 progeny that were homozygous for both *sma-9* and the suppressor mutation were collected. F3 generation worms from these F2 progeny were pooled for DNA extraction and library construction. Worm genomic DNA was prepared using the Qiagen Gentra Puregene Kit. 5μg of genomic DNA was used to prepare the sequencing library using the NEBNext DNA Sample Prep Master Mix Set 1. Single-end 50bp short-read (51 cycle) sequencing was performed on the HiSeq 2000 instrument (Illumina), yielding 38 ~ 78 million reads (20 ~ 41 fold coverage) per sample.

For direct WGS (*jj58*, *jj60*, *jj61*, *jj71*, *jj77*), data analysis was done using the MAQGene platform [86, 87] with the default setting. SNP variants on the X chromosome compared to the reference *C*. *elegans* genome ce6 W221 were analyzed. Genes with missense SNP variants in *jj60* and *jj77*, but not in *jj58* and *jj71*, were among the candidate genes that were targeted by RNAi for their ability to suppress the *sma-9(cc604)* M lineage defects by injection. These included C41A3.1, K09C4.8 and C17G1.8. Further PCR and sequencing confirmed the *jj60* and *jj77* mutations in C17G1.8 (*tsp-21*).

For mapping additional suppressors using either direct WGS (for *jj2*, *jj5*, *jj7*, *jj50*, *jj52* and *jj70*) or SNP-WGS (for *jj49*, *jj57*, *jj62*, *jj69*, *jj71*, *jj73*, *jj78* and *jj83*), sequence data were aligned to *C*. *elegans* reference genome version WS220 using BFAST [88] with default parameters. SNP calling was performed by SAMTOOLS [89]. A valid SNP call required a minimum read depth of three. ANNOVAR [90] was used for annotation of SNP coding potential. For SNP-WGS, Hawaiian SNPs were annotated with a custom Perl script. Scatter plots of heterozygous (0.2–0.7 fraction of total reads) Hawaiian SNPs were generated as chromosome position vs. fractional total graphs. Mapping intervals were defined by visual inspection for gaps (i.e., Hawaiian SNP fraction <0.2). Candidate suppressor genes were identified as homozygous (fraction >0.8), non-Hawaiian, nonsynonymous SNPs in the mapped interval. The SNPs in the identified suppressor genes were verified by PCR and sequencing.

*jj61* was mapped via SNP-WGS to the region on the X chromosome where *lon-2* is located. Direct inspection of the sequence reads around the *lon-2* region showed that *jj61* contains a large deletion (11.8kb) spanning the *lon-2* region, which was subsequently verified by PCR and sequencing.

### Plasmid constructs and transgenic lines

*sma-6* reporter and rescuing constructs

pJKL840: *sma-6p*::*nls*::*rfp*::*lacZ*::*unc-54 3’UTR*

pJKL1048: *sma-6(jj69)p*::*nls*::*rfp*::*lacZ*::*unc-54 3’UTR*

pJKL1060: *sma-6p*::*sma-6* rescuing construct

*tsp-21* reporter constructs

pJKL1005: *5kb tsp-21p*::*tsp-21 genomic ORF*::*1*.*7kb tsp-21 3’UTR*

pJKL1004: *5kb tsp-21p*::*tsp-21 genomic ORF*::*gfp*::*1*.*7kb tsp-21 3’UTR*

pJKL998: *5kb tsp-21p*::*nls*::*gfp*::*lacZ*::*unc-54 3’UTR*

pZL11: *5kb tsp-21p*::*tsp-21 genomic ORF (sgRNA target site modified)*::*gfp*::*1*.*7kb tsp-21 3’UTR*

Constructs for tissue-specific expression of *tsp-21*

pJKL1015: *tsp-21p*::*tsp-21 cDNA*::*tsp-21 3’UTR*

pJKL1017: *rol-6p*::*tsp-21 cDNA*::*tsp-21 3’UTR*

pJKL1018: *elt-3p*::*tsp-21 cDNA*::*tsp-21 3’UTR*

pJKL1019: *elt-2p*::*tsp-21 cDNA*::*tsp-21 3’UTR*

pJKL1020: *hlh-8p*::*tsp-21 cDNA*::*tsp-21 3’UTR*

pJKL1021: *myo-2p*::*tsp-21 cDNA*::*tsp-21 3’UTR*

The full-length *tsp-21* cDNA clone *yk1449c02*, which contains a SL1 trans-splice leader sequence, and full length 5’ and 3’ UTRs, was kindly provided by Dr. Yuji Kohara (National Institute of Genetics, Japan). A point mutation in the coding region of *tsp-21* in *yk1449c02* was corrected by site-directed mutagenesis to generate pJKL994. Transgenic animals were generated using the plasmid pRF4 or pJKL449 (*myo-2p*::*gfp*::*unc-54 3’UTR*) as markers. Integrated transgenic lines carrying pJKL1004[TSP-21::GFP] (*jjIs3113* and *jjIs3114*) were generated using gamma-irradiation. pJKL840[*sma-6p*::*nls*::*rfp*::*lacZ*::*unc-54 3’UTR*] was used for co-localization of TSP-21::GFP and *sma-6p*::*nls*::*rfp*. pTAA1[*hlh-8p*::*cgt-3*.*1a ORF*::*unc-54 3’UTR*] was used to test for function of *cgt-3* in the M lineage.

### Generating TSP-21::GFP using CRISPR technology

The following mix of plasmid DNAs was injected into the N2 gravid adults: (1) a Cas9 expression plasmid pDD162 [91], (2) a *tsp-21*-specific sgRNA plasmid pZL10, which has GAAACTGACACGGTAGAAGATGG replacing the *unc-119* sgRNA in plasmid 46169 [92], (3) the homologous repair template pZL11: *5kb tsp-21p*::*tsp-21 genomic ORF (sgRNA target site modified)*::*gfp*::*1*.*7kb tsp-21 3’UTR*, (4) a co-injection marker pCFJ90[*myo-2p*::*mCherry]* [93]. GFP knock-in events were screened via PCR using a primer in GFP (ZL21: CGCATATCTTGGACGCCTAATTTG) and a primer in the *tsp-21* 3’ region outside of the sequences included in pZL11 (ZL22: TCCACACAATCTGCCCTTTCG). Single worm PCR of 250 F1s failed to detect any germline integration event. However, we checked the F2 generation for high transmission efficiency lines (*myo-2*::*mCherry* positive) and screened via PCR 5–10 F3 progeny from each of the three high transmission efficiency lines (>50%). One of the three transgenic lines gave us two homozygous GFP knock-in strains: LW3670: *jj93(tsp-21*::*gfp)* and LW3671: *jj94(tsp-21*::*gfp)*.

### RT-PCR

Total RNA was isolated from mixed-stage N2 or *sma-6(jj69)* worms using TRIzol Reagent (Invitrogen). Reverse transcription was performed with SuperScript III First-Strand Synthesis System (Invitrogen) following the manufacturer’s instructions. The primers used to detect the cDNAs of *sma-6* and *act-1* are: *sma-6*, MLF-34 and MLF-44; *act-1*, NMA-163 and NMA-164.

### Body size measurement, RAD-SMAD reporter assay and dauer formation assay

Body size measurement and RAD-SMAD reporter assay were carried out as described in Tian et al. [22]. Dauer formation assay was carried out as described in Tian et al. [21]. Statistical analyses were performed using Microsoft Excel and GraphPad Prism (http://www.graphpad.com/scientific-software/prism/).

### Fluorescence microscopy

GFP and RFP epifluorescence in transgenic animals was visualized either on a Leica DMRA2 compound microscope, where the images were captured by a Hamamatsu Orca-ER camera using the OPENLAB software, or on a Zeiss LSM 710 confocal microscope. Subsequent image analysis was performed using ImageJ and Photoshop CC.

### Tetraspanin phylogenetic analysis

We identified tetraspanin homologs by running hmmsearch from HMMER 3.1b1 [94] with the hidden Markov model (HMM) profile for tetraspanins (PF00335.15) from PFAM 27.0 [95] against the reference proteome set of the Quest for Orthologs consortium ([96]; source URL, *ftp*:*/ftp*.*ebi*.*ac*.*uk/pub/databases/reference_proteomes/QfO/QfO_release_2014_04*.*tar*.*gz*). hmmsearch was run with the arguments '*-E 1e-06—domE 1e-06—incE 1e-06—incdomE 1e-06-A [alignment]*', which generated aligned regions of similarity to these core domains. Since the regions of homology were extracted from full-length proteins with an HMM, the specific residues extracted were generally a subset of the full protein; moreover, it was possible for two or more such regions to be independently extracted from a single protein chain, although this proved rare for tetraspanins.

These regions were then realigned with MAFFT v7.158b [97] in L-INS-i, its slowest and most reliable mode, using the arguments '*—localpair—maxiterate 1000*'. The resulting alignments were purged of poorly aligned members by first running trimal v1.4.rev15 [98] using the argument '*-gt 0*.*5*', and then running BMGE 1.1 [99] using the arguments '*-t AA-h 1-g 0*.*5*:*1*'. This purged the alignments of any columns in which over 50% of the columns' positions consisted of gaps rather than amino acid residues, and then any sequences in which over 50% of the residues were gapped, yielding global alignments that lacked excessive loops and gaps. From the filtered alignments, we computed protein maximum-likelihood phylogenies, with a WAG model of amino acid evolution [100] and with pseudocounts for gaps, via FastTree 2.1.7 [101], using the arguments '*-pseudo-wag*'. Confidence values for the branches of trees (ranging from 0.00 to 1.00) were automatically computed by FastTree with 1,000 internal replicates. We visualized the resulting trees with FigTree 1.4.2 (*http://tree.bio.ed.ac.uk/software/figtree*). Branch lengths were measured in average substitutions (when comparing full sequences or their profiles) among non-gap positions in the aligned sequences, with distances derived from the BLOSUM45 matrix, a correction for multiple substitutions, and an allowed maximum of 3.0 substitutions per individual site [102].

### Split-ubiquitin yeast two-hybrid analysis

The split-ubiquitin yeast two-hybrid experiments were carried out following the detailed method described in Grefen et al. [103]. The bait CubPLV and prey NubG constructs were generated via PCR and recombinational in vivo cloning in yeast [103]. The resulting fusion constructs were recovered from yeast and transformed into *E*. *coli* and confirmed by sequencing. The primers, cDNA templates, and the names of the resulting bait and prey constructs are summarized in S2 Table. The bait and prey constructs were transformed into the haploid yeast strains THY.AP4 (MATa) and THY.AP5 (MATα), respectively, and the resulting yeast strains were mated to generate diploid yeast cells carrying specific combinations of bait and prey constructs [103]. Interactions among each pair of bait and prey constructs were visualized by streaking diploid cells on SC-Trp,-Leu,-Ade,-His,-Ura,-Met plates that were supplemented with four different concentrations of methionine: 0mM, 0.075mM, 0.150mM and 0.300mM, respectively. Methionine can repress the expression of the CubPLV fusion, which is under the control of the Met-repressible *MET25* promoter [42]. Growth was monitored for 2–9 days at 30°C.

The plasmids KAT-1-Cub-PLV, NubG-KAT-1 (in pNX33 vector) and KAT-1-NubG (in pXN21 vector) [42], HMT-1-Cub-PLV and HMT-1-NubG (in pXN21 vector) [46] were kindly provided by Sungjin Kim (Cornell University) and used as specificity controls. NubG fusions for PAR-4, a protein unexpected to interact with any of the proteins tested, was included as another control for specificity of the interactions. The empty NubG vector was used as a control to determine if any Cub-PLV fusions can auto-activate the reporters. The vector expressing soluble wild-type Nub (NubWT) was used as a control to indicate expression of the Cub-PLV fusion. Additional confirmation of expression of each fusion protein came from western blot analysis using rabbit polyclonal anti-VP16 antibodies (ab4808, Abcam, for CubPLV fusions) and monoclonal anti-HA antibodies (Clone 12CA5, Sigma, for NubG fusions).

## Supporting Information

S1 Fig Maximum likelihood phylogeny of tetraspanin homologs. Identities of the sequences are given in Supplemental Table 4. Confidence values (posterior probabilities) for branches of a phylogeny are given as decimal fractions. Branch lengths are measured in average substitutions among non-gap positions. The *C*. *elegans* tetraspanin proteins are colored in red, while their corresponding human homologs are colored in blue. Each subtree (clade) containing one or more *C*. *elegans* tetraspanin genes is displayed on one page and shaded in a different color. The human orthologs of *tsp-21* are TSPAN4, TSPAN9, and CD53; while the human orthologs of *tsp-12* and *tsp-14* are TSPAN5, TSPAN10, TSPAN14, TSPAN15, TSPAN17 and TSPAN33.
(PDF)

S2 Fig The M lineage phenotypes in different mutants that affect M lineage dorsoventral patterning. Schematic representation of the M lineage (A-D) and diagrams showing the CC locations (E-H) in wild-type or *sma-9(0);susm* (A, E), *sma-9(0)* (B, F), *lin-12(0)* (C, G), and *lin-12(0);sma-9(0)* (D, H) animals. BWM: body-wall muscle, CC: coelomocyte, SM: sex myoblast. d: dorsal, v: ventral, l: left, r: right, a: anterior, p: posterior. M lineage-derived CCs are marked by blue arrowheads.
(PDF)

S3 Fig A simplified glycosphingolipid metabolic pathway in *C*. *elegans*. An abbreviated glycosphingolipid metabolic pathway (based on [52]) showing the genes (red) and their corresponding enzymes (blue) that we tested in this work.
(PDF)

S1 Table *tsp-21(jj77)* has no effect on the Daf-c phenotypes of *daf-1* and *daf-7* mutants. (DOCX)

S2 Table Summary of the reagents used for the split-ubiquitin yeast two-hybrid assay. (DOCX)

S3 Table The locations of M-derived CCs in *cgt-3(ok2877); sma-9(cc604)* mutants support a role of *cgt-3* in both Sma/Mab and LIN-12/Notch signaling. (DOCX)

S4 Table List of 700 tetraspanin homologs. The sequence names of 700 tetraspanin homologs phylogenetically analyzed in S1 Fig are listed, along with their species of origin, their accession identifier names, and the amino acid residues from the full-length homolog that were aligned and used for the phylogenetic analysis. The accession names are from the SwissProt and TrEMBL divisions of UniProt (indicated by "sp|" and "tr|"), via the reference proteome set of the Quest for Orthologs consortium.
(XLSX)
