# Supplementary figures and images for: Promotion of Bone Morphogenetic Protein Signaling by Tetraspanins and Glycosphingolipids
Source: PLoS Genet. 2015 May 15;11(5):e1005221. doi: 10.1371/journal.pgen.1005221 (PMC4433240; doi:10.1371/journal.pgen.1005221)

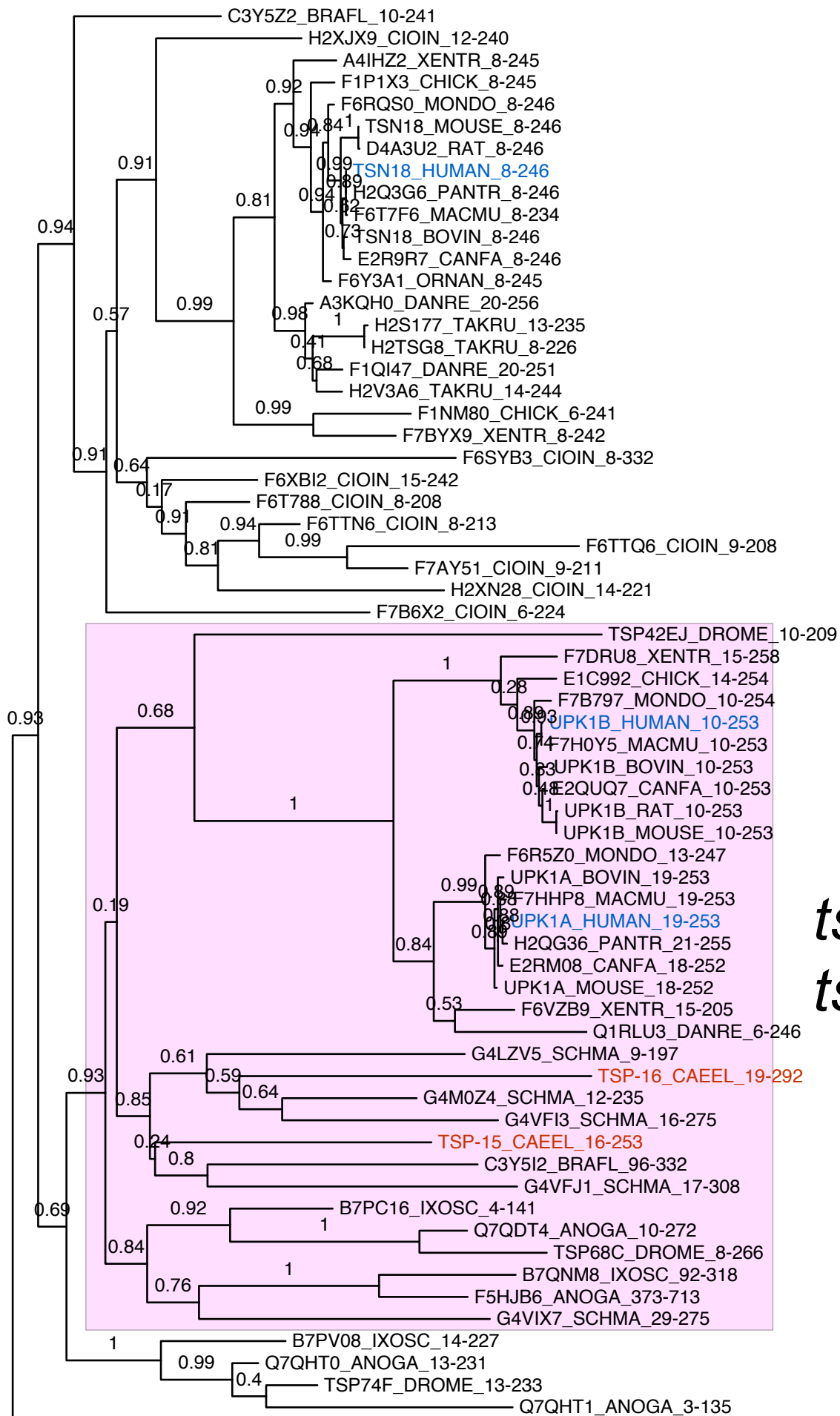

*tsp-15,*  
*tsp-16*

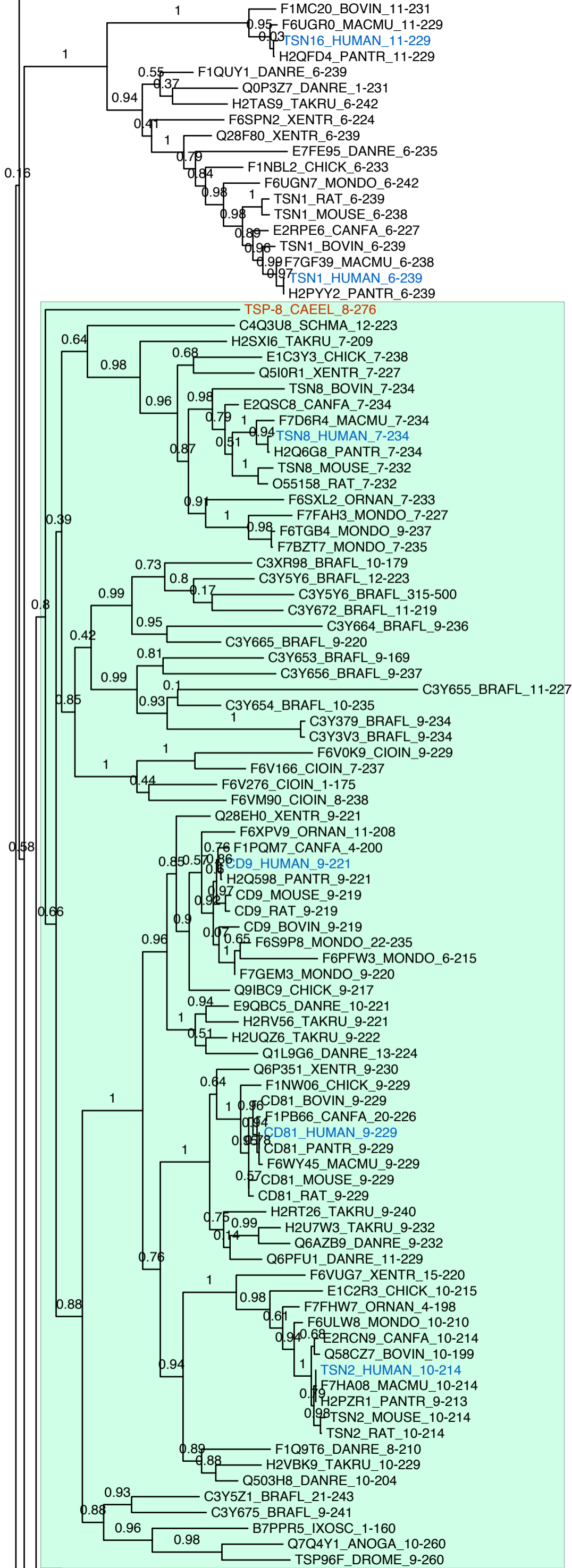

*tsp-8*

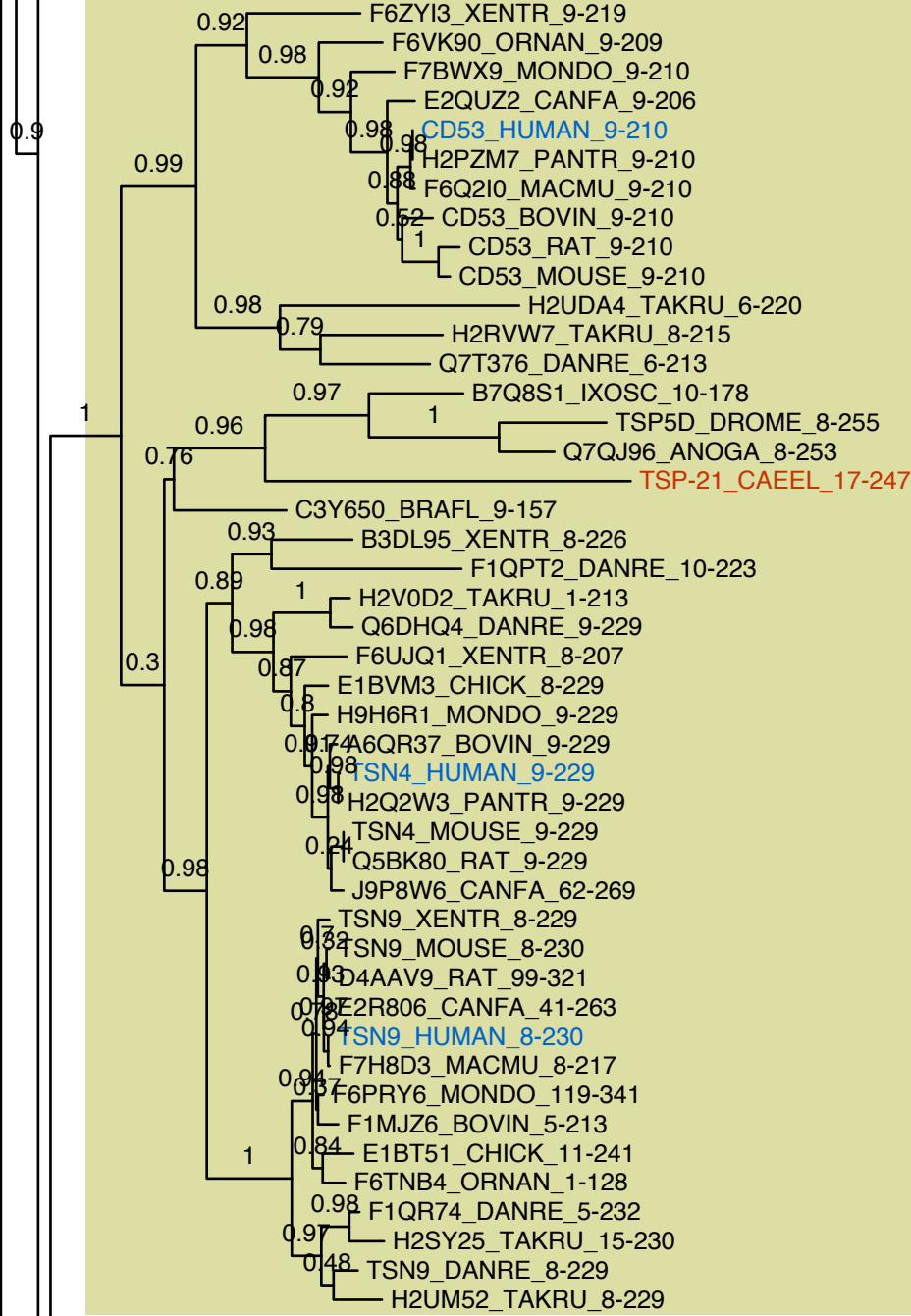

*tsp-21*

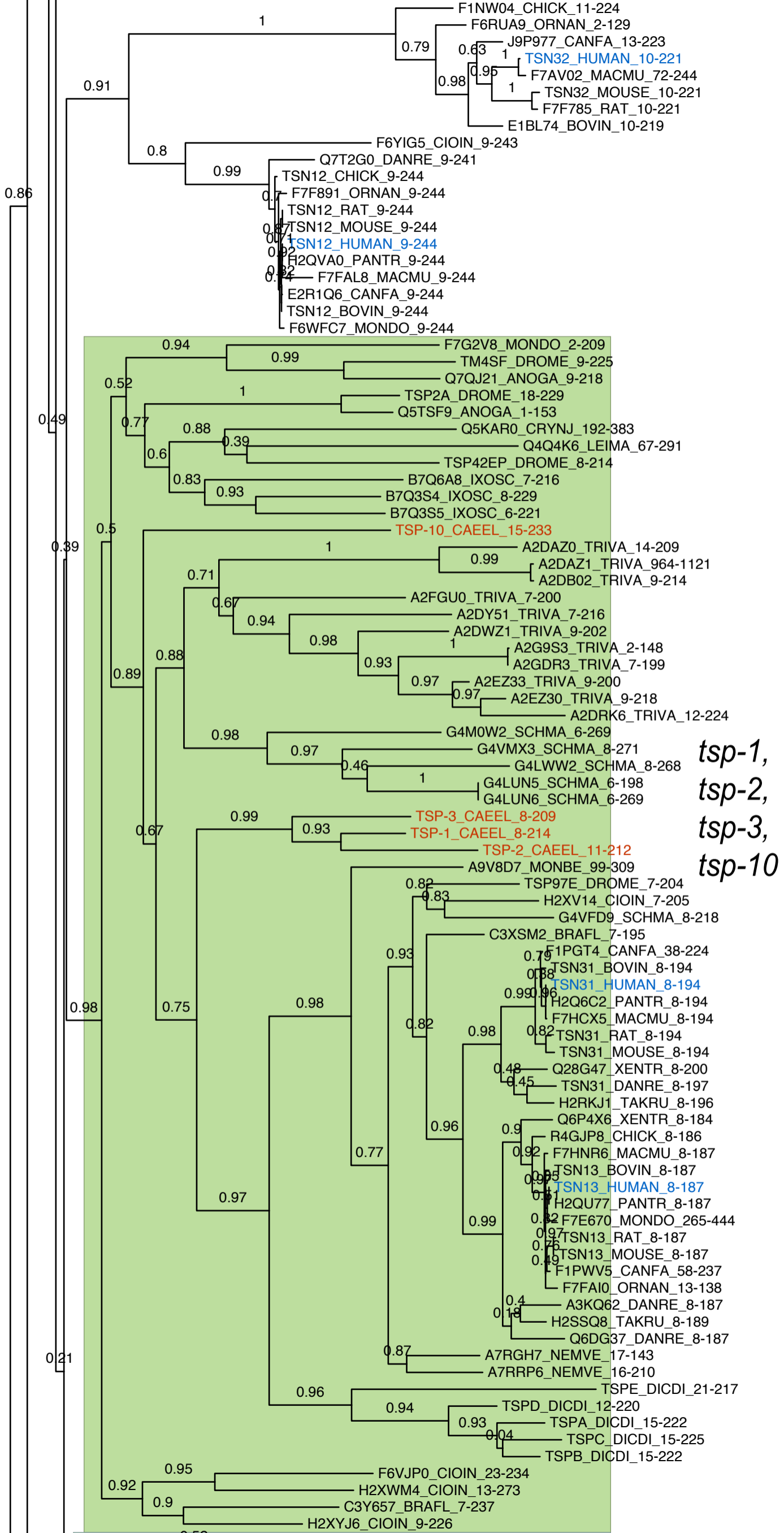

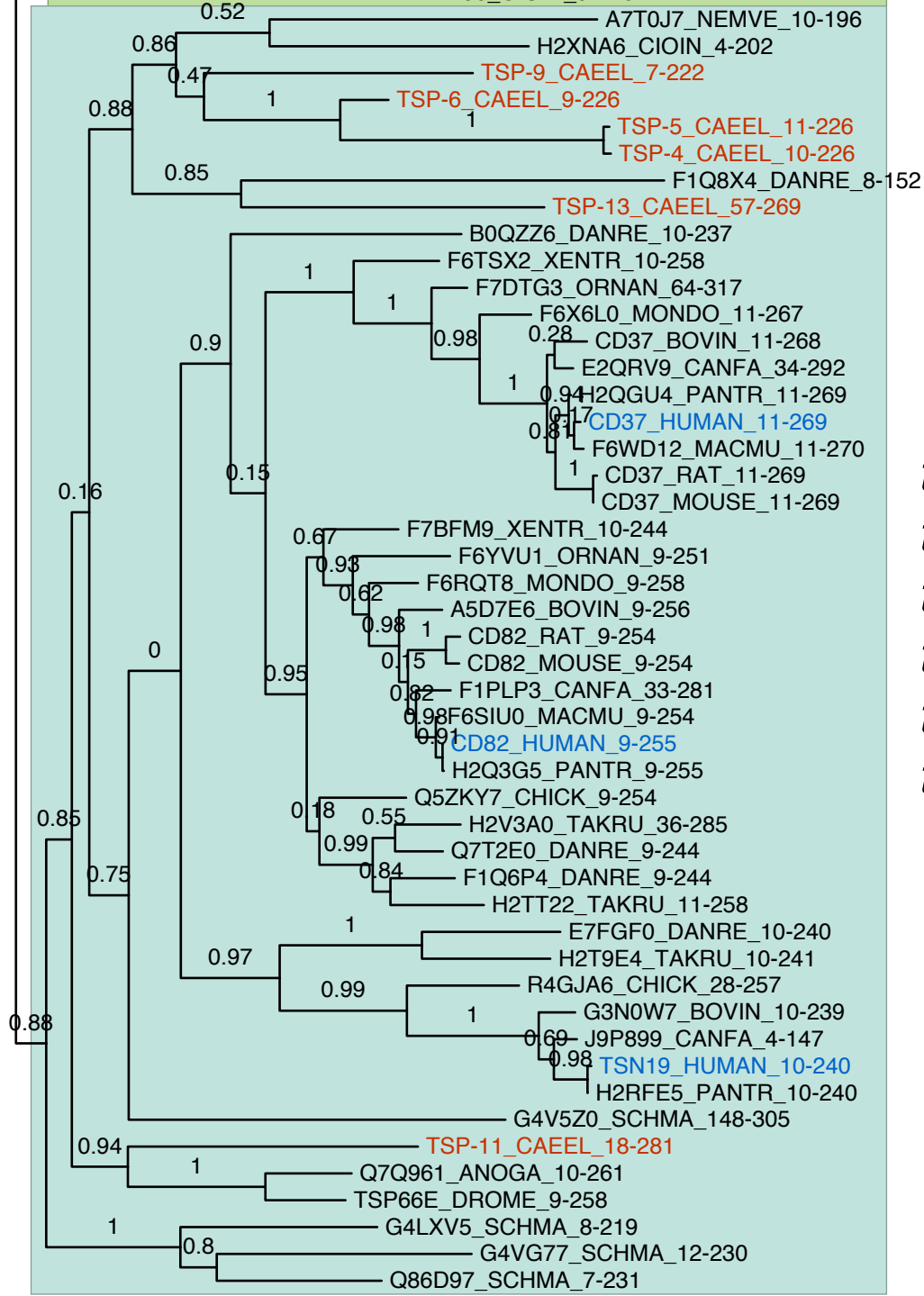

*tsp-4,*  
*tsp-5,*  
*tsp-6,*  
*tsp-9,*  
*tsp-11,*  
*tsp-13*



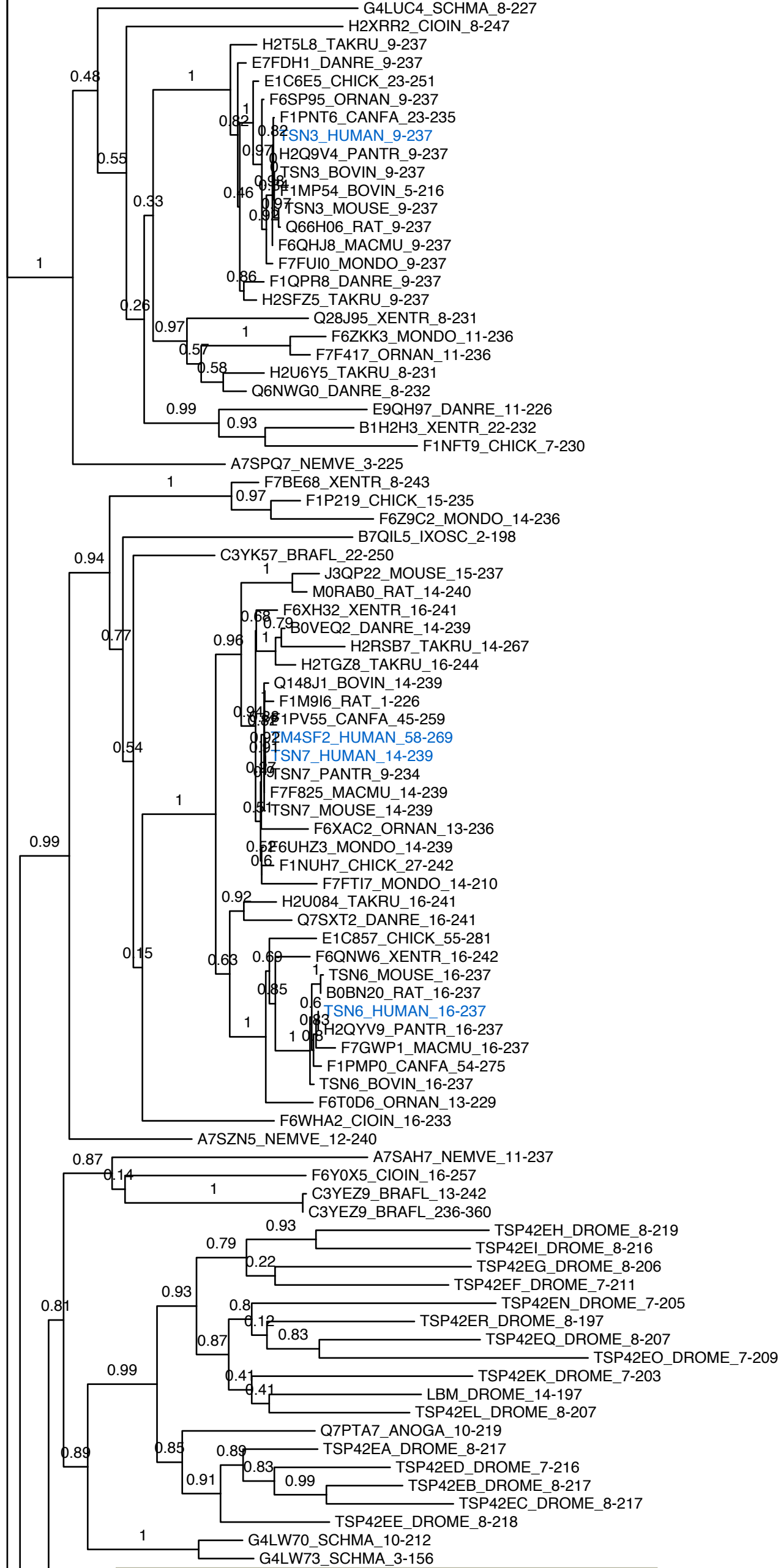

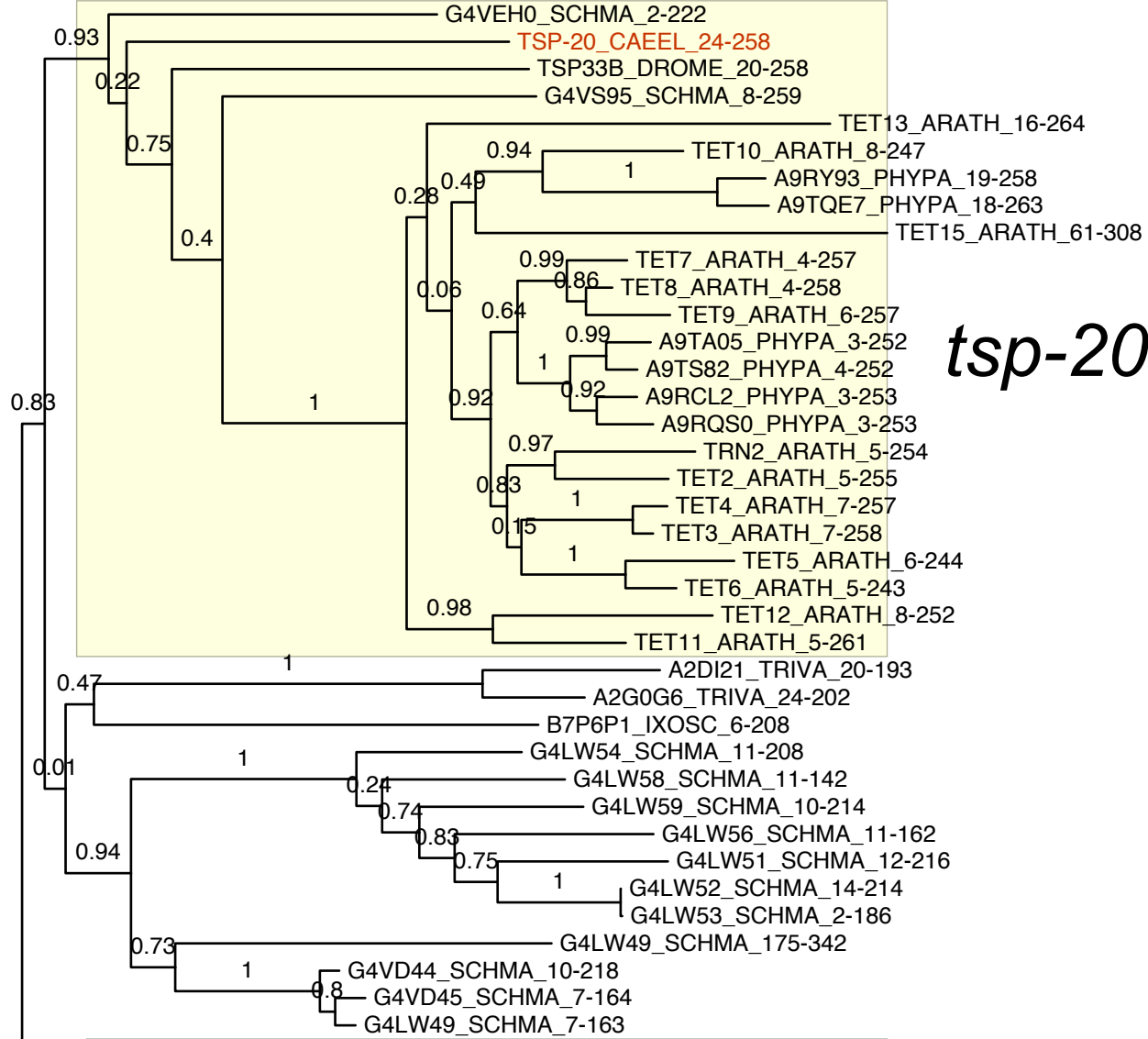

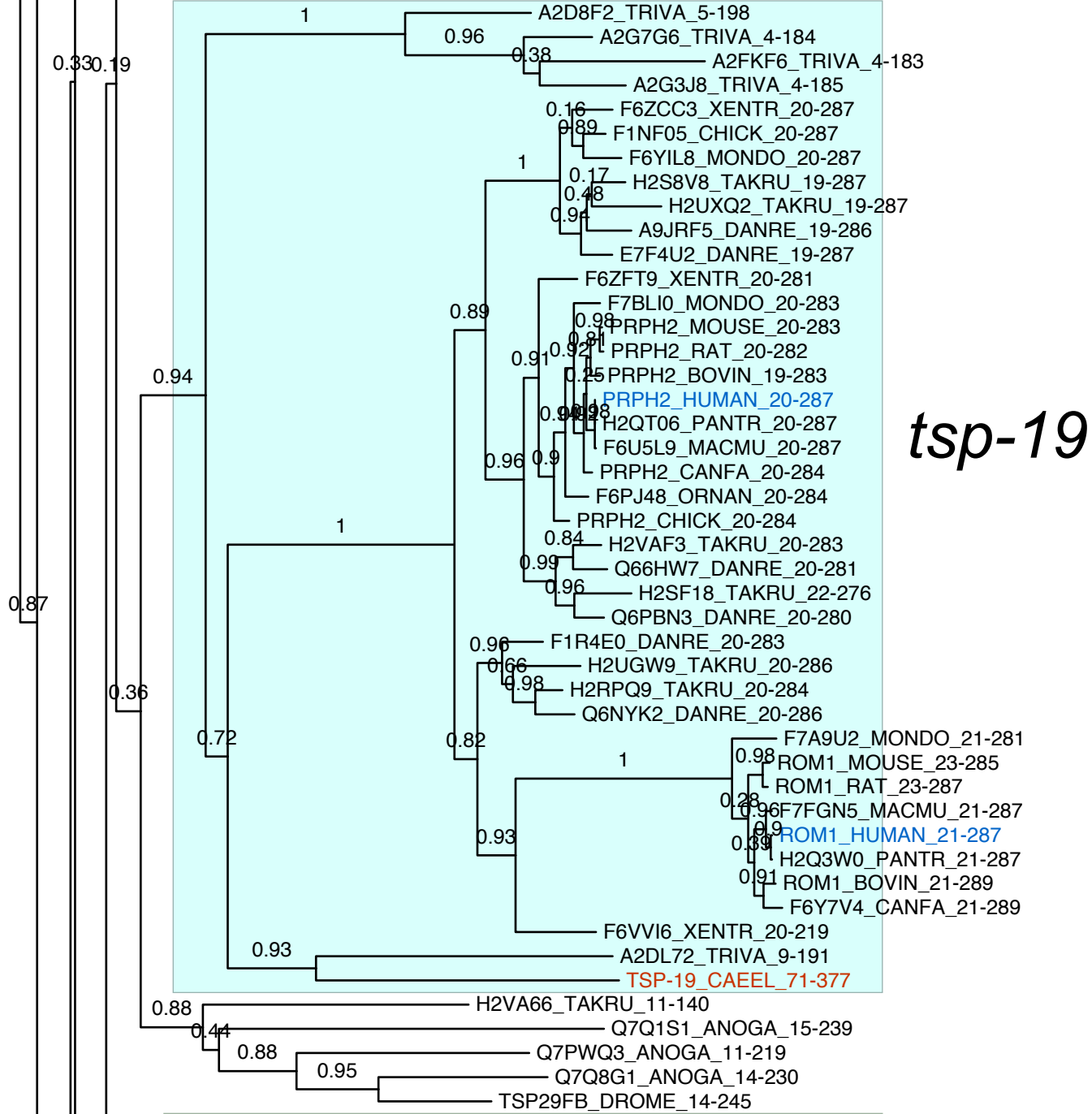

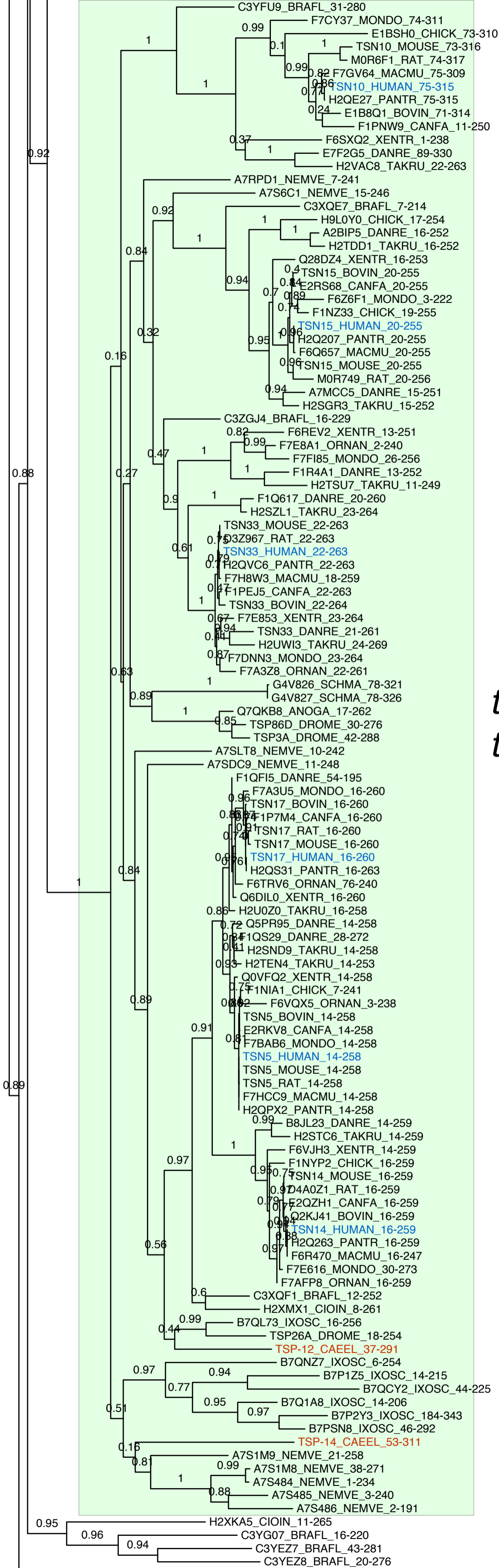

*tsp-12,*  
*tsp-14*

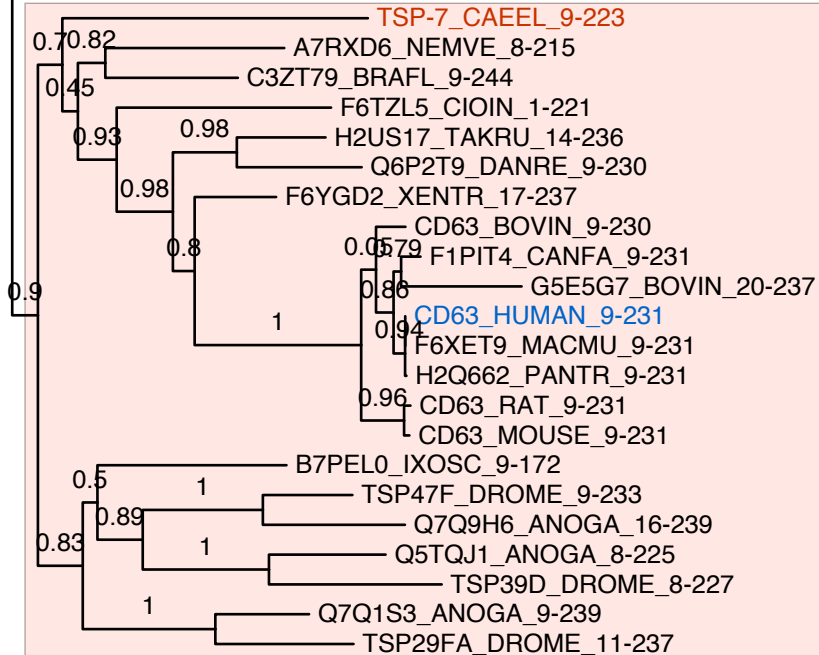

*tsp-7*

0.5

Supplement: S1 Fig — Identities of the sequences are given in Supplemental Table 4. Confidence values (posterior probabilities) for branches of a phylogeny are given as decimal fractions. Branch lengths are measured in average substitutions among non-gap positions. The C. elegans tetraspanin proteins are colored in red, while their corresponding human homologs are colored in blue. Each subtree (clade) containing one or more C. elegans tetraspanin genes is displayed on one page and shaded in a different color. The human orthologs of tsp-21 are TSPAN4, TSPAN9, and CD53; while the human orthologs of tsp-12 and tsp-14 are TSPAN5, TSPAN10, TSPAN14, TSPAN15, TSPAN17 and TSPAN33. (PDF) [file pgen.1005221.s001.pdf]

Wild type or *sma-9(0); susm*

*sma-9(0)*

*lin-12(0)*

*lin-12(0); sma-9(0)*

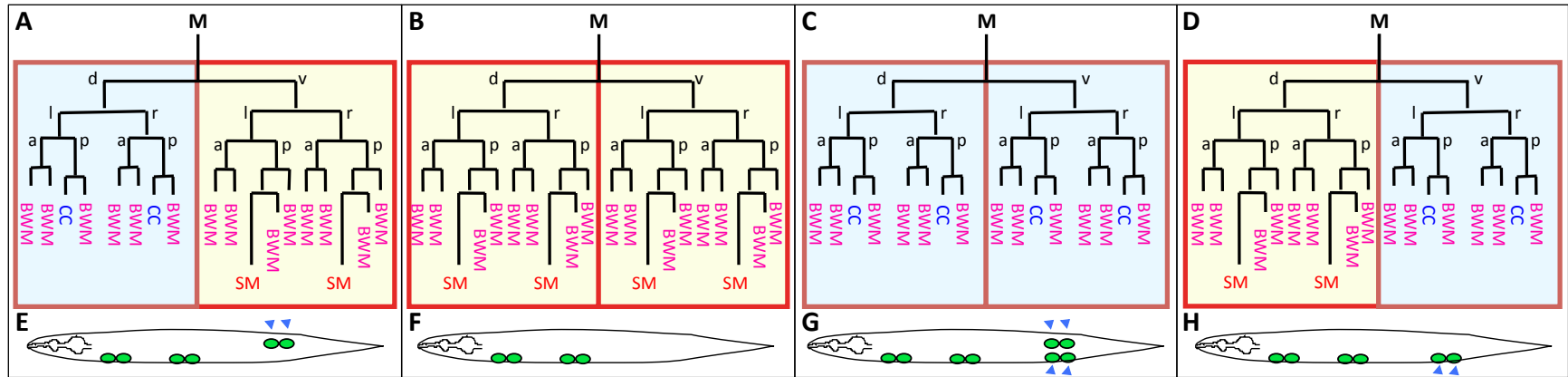

Supplement: S2 Fig — Schematic representation of the M lineage (A-D) and diagrams showing the CC locations (E-H) in wild-type or sma-9(0);susm (A, E), sma-9(0) (B, F), lin-12(0) (C, G), and lin-12(0);sma-9(0) (D, H) animals. BWM: body-wall muscle, CC: coelomocyte, SM: sex myoblast. d: dorsal, v: ventral, l: left, r: right, a: anterior, p: posterior. M lineage-derived CCs are marked by blue arrowheads. (PDF) [file pgen.1005221.s002.pdf]

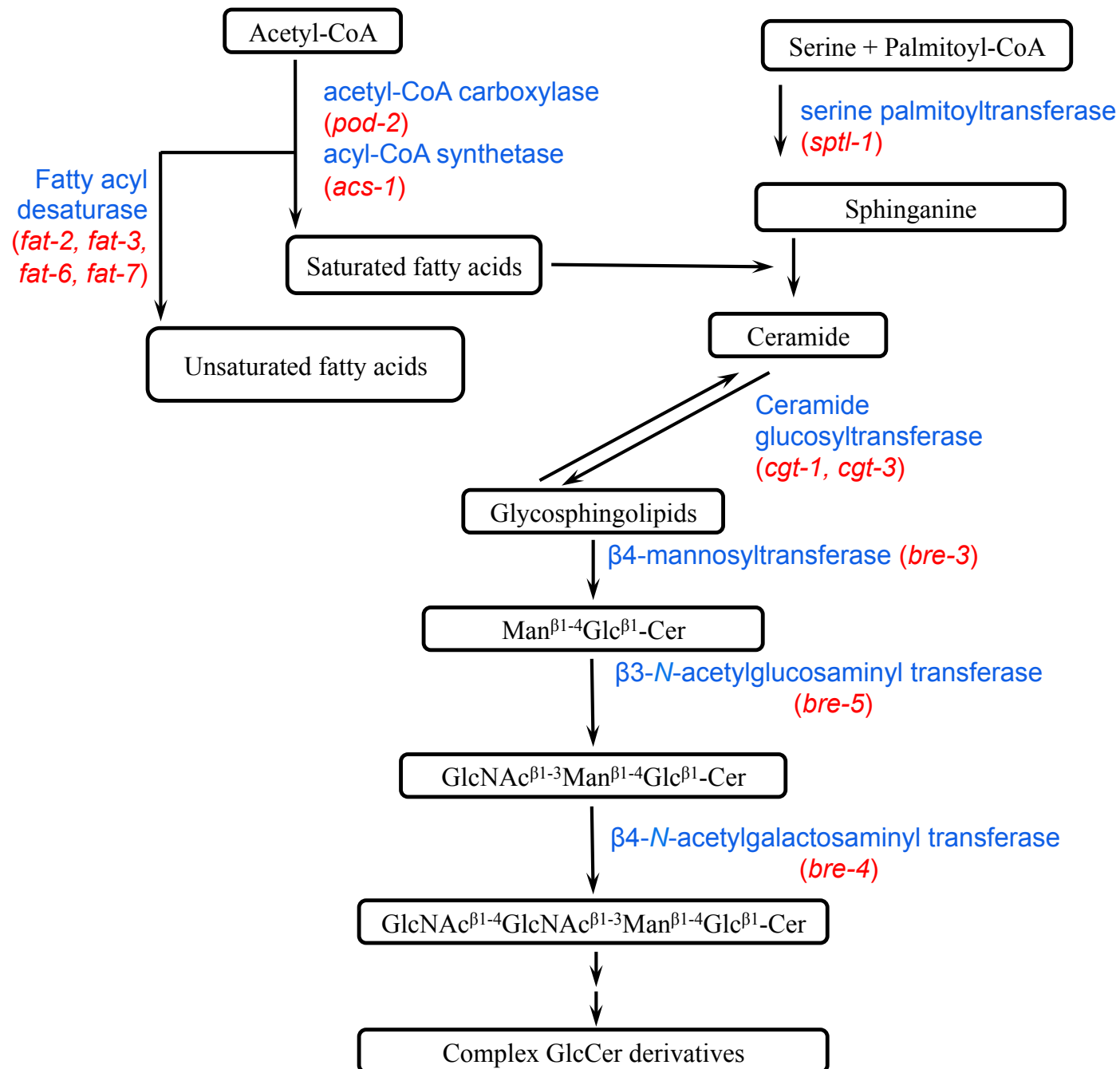

Supplement: S3 Fig — An abbreviated glycosphingolipid metabolic pathway (based on [52]) showing the genes (red) and their corresponding enzymes (blue) that we tested in this work. (PDF) [file pgen.1005221.s003.pdf]
