# Supplementary material for: Promotion of Bone Morphogenetic Protein Signaling by Tetraspanins and Glycosphingolipids
Source: PLoS Genet. 2015 May 15;11(5):e1005221. doi: 10.1371/journal.pgen.1005221 (PMC4433240; doi:10.1371/journal.pgen.1005221)
Supplement: S1 Table — (DOCX) [file pgen.1005221.s004.docx]

**Supplemental table 1. *tsp-21(jj77)* has no effect on the Daf-c phenotypes of *daf-1* and *daf-7* mutants**

|  | **15°C** | | | **20°C** | | | **25°C** | | |
| --- | --- | --- | --- | --- | --- | --- | --- | --- | --- |
|  | *n* | % Daf-c (pop) | *t*-value | *n* | % Daf-c (pop) | *t*-value | *n* | % Daf-c (pop) | *t*-value |
| *tsp-21(jj77-898)** | 4 | 0 (734) | -- | 3 | 0 (582) | -- | 3 | 0 (608) | -- |
| *tsp-21(jj77-778)** | 4 | 0 (715) | -- | 3 | 0 (348) | -- | 3 | 0 (286) | -- |
| *daf-1(m40)* | 5 | 0.2±0.5 (841) | -- | 5 | 7.9±3.6 (950) | -- | 5 | 100 (969) | -- |
| *daf-1(m40); tsp-21(jj77-898)* | 5 | 0.7±0.9 (280) | 0.45 (3.36) | 5 | 13.6±6.0 (471) | 0.81 (3.36) | 4 | 100 (338) | -- |
| *daf-1(m40); tsp-21(jj77-778)* | 5 | 0.4±1.0 (223) | 0.19 (3.36) | 5 | 13.2±5.3 (756) | 0.83 (3.36) | 5 | 100 (505) | -- |
| *daf-1(m213)* | 5 | 0.4±0.4 (677) | -- | 5 | 69.8±5.9 (906) | -- | 4 | 100 (865) | -- |
| *daf-1(m213); tsp-21(jj77-898)* | 5 | 0.8±1.1 (238) | 0.34 (3.36) | 4 | 66.3±14.0 (466) | 0.23 (3.50) | 4 | 100 (422) | -- |
| *daf-1(m213); tsp-21(jj77-778)* | 5 | 0.3±0.8 (359) | 0.19 (3.36) | 5 | 73.3±4.7 (591) | 0.46 (3.36) | 4 | 100 (407) | -- |
| *daf-7(m62)* | 5 | 64.3±7.1 (255) | -- | 9 | 86.9±11.1 (1259) | -- | 4 | 100 (776) | -- |
| *daf-7(m62); tsp-21 (jj77-898)* | 6 | 73.9±6.8 (333) | 0.98 (3.25) | 10 | 77.3±7.4 (718) | 0.72 (2.90) | 4 | 100 (831) | -- |

** 898* and *778* are two independent *jj77* recombinants as described in Materials and Methods.

*n*, the number of plates scored at each temperature.

% Daf-c, the weighted mean and standard deviation of dauer formation percentages for *n* plates.

pop, total number of worms on *n* plates scored.

*t*-value, as calculated by Student’s *t-*test, between *daf* single mutants and the corresponding double mutants at the specified temperature. Minimum *t*-values required for 99% significance level are indicated in parentheses. The each *t-*test, the *t*-value is significantly lower than the corresponding minimum *t*-value required for 99% significance level.
