# Supplementary material for: Promotion of Bone Morphogenetic Protein Signaling by Tetraspanins and Glycosphingolipids
Source: PLoS Genet. 2015 May 15;11(5):e1005221. doi: 10.1371/journal.pgen.1005221 (PMC4433240; doi:10.1371/journal.pgen.1005221)
Supplement: S2 Table — (DOCX) [file pgen.1005221.s005.docx]

**Supplemental table 2. Summary of the reagents used for the split-ubiquitin yeast two-hybrid assay.**

| **Gene** | **Primer pairs** | **cDNA template** | **X-CubPLV**^e^ | **NubG-X(32)**^e^ |
| --- | --- | --- | --- | --- |
| *tsp-21* | NAS5, NAS6 | pJKL994^a^ | pNAS7 | pNAS33 |
| *tsp-12* | NAS3, NAS4 | 10025@B4^b^ | pNAS1 | pNAS27 |
| *tsp-14* | NAS1, NAS2 | 11081@G03^b^ | pNAS4 | pNAS30 |
| *sma-6* | NAS11, NAS12 | yk786c02 | pNAS16 | pNAS42 |
| *daf-4* | JKL1292, JKL1293 | pJKL1051^c^ | pJKL1052 | pJKL1054 |
| *par-4* | JKL1302, JKL1303 | LiuFD243^d^ | - | pJKL1062 |

^a^ See Materials and Methods.

^b^ From the Vidal RNAi library [82].

^c^ Full length *daf-4* a cDNA generated by using *yk1045b09* and *yk1068e07*.

^d^ Full length *par-4* cDNA in the zero-blunt vector, kind gift of Diane Morton [45].

^e^ X refers to protein of interest. All the vectors are described in Grefen et al. [103].
