# Supplementary material for: Promotion of Bone Morphogenetic Protein Signaling by Tetraspanins and Glycosphingolipids
Source: PLoS Genet. 2015 May 15;11(5):e1005221. doi: 10.1371/journal.pgen.1005221 (PMC4433240; doi:10.1371/journal.pgen.1005221)
Supplement: S3 Table — (DOCX) [file pgen.1005221.s006.docx]

**Supplemental table 3. The locations of M-derived CCs in *cgt-3(ok2877); sma-9(cc604)* mutants support a role of *cgt-3* in both Sma/Mab and LIN-12/Notch signaling**

| Number and location  of M-derived CCs | *cgt-3(ok2877); sma-9(cc604)* isolates | |
| --- | --- | --- |
|  | Isolate 19.1 (n=260) | Isolate 5.1 (n=132) |
| 0 CC | 50.00% | 65.91% |
| 1 CC (dorsal) | 31.15% | 23.48% |
| 1 CC (ventral) | 11.15% | 3.79% |
| 2 CC (both dorsal) | 5.77% | 4.55% |
| 2 CC (both ventral) | 0.38% | 0.00% |
| 2 CC (1 ventral + 1 dorsal) | 1.15% | 1.52% |
| 3 CC (all dorsal) | 0.38% | 0.76% |
